# Therapeutic Advances in Oncology

**DOI:** 10.3390/ijms22042008

**Published:** 2021-02-18

**Authors:** Jinsha Liu, Priyanka Pandya, Sepideh Afshar

**Affiliations:** Protein Engineering, Lilly Biotechnology Center, Eli Lilly and Company, San Diego, CA 92121, USA; liu_jinsha@lilly.com (J.L.); pandya_priyanka@lilly.com (P.P.)

**Keywords:** therapeutic modalities, innovation, oncology, breast cancer, lung cancer, multiple myeloma

## Abstract

Around 77 new oncology drugs were approved by the FDA in the past five years; however, most cancers remain untreated. Small molecules and antibodies are dominant therapeutic modalities in oncology. Antibody-drug conjugates, bispecific antibodies, peptides, cell, and gene-therapies are emerging to address the unmet patient need. Advancement in the discovery and development platforms, identification of novel targets, and emergence of new technologies have greatly expanded the treatment options for patients. Here, we provide an overview of various therapeutic modalities and the current treatment options in oncology, and an in-depth discussion of the therapeutics in the preclinical stage for the treatment of breast cancer, lung cancer, and multiple myeloma.

## 1. Introduction

More than 17 million new cancer cases have been reported worldwide in the past two years and the number is expected to grow to 27.5 million by 2040 [1]. Investment in early detection methods, awareness of carcinogens and, most importantly, the development of novel therapeutics has resulted in decreased mortality in cancer patients. Around 77 new oncology therapeutics were approved in the last five years (Table S1), however, oncology still remains as one of the most challenging areas of drug discovery [2]. 

### 1.1. Small Molecule Therapeutics

Small molecules remain the major therapeutic modalities for cancer treatment (Figure S1). Since 2015, 52 novel small molecule cancer drugs have been approved (Table 1). To increase specificity and potency, majority of these small molecules are defying Lipinski’s Rule of 5 (Ro5) [3]; their molecular weight exceeds 500 Da and they have higher number of hydrogen bond donor and acceptors than the traditional small molecules. As a result, they are more potent but their permeability, solubility, and metabolic clearance are negatively impacted. Nonetheless, it was speculated that the high potency and specificity might compensate for the poor bioavailability and converge into reasonable oral doses [4]. The novel bifunctional small molecules called PROTACs (proteolysis-targeting chimeras) exploit ubiquitin-proteasome system (UPS) machinery to degrade disease-causing intracellular proteins [5]. The first two orally bioavailable PROTACs, ARV-110, and ARV-471, target androgen receptor for prostate cancer and estrogen receptor for breast cancer, respectively, and are in clinic since 2019 [6,7].

### 1.2. Large Molecule Therapeutics

Monoclonal antibodies (mAbs) have emerged as a novel approach in oncology due to their high affinity and specificity. The first therapeutic mAb Orthoclone OKT3 (muromonab-CD3), produced in mouse cells, was approved in 1986 for the prevention of kidney transplant rejection. By September 2020, about 80 oncology-related mAbs were in the market with twelve approved in the past five years (Table 2). Remarkable advancements in antibody discovery and computational platforms have facilitated development of humanized/human mAbs with high potency, improved developability, and decreased immunogenicity. Currently, mAbs are considered as the main modalities for targeted therapy and immunotherapy (e.g., targeting CTLA4 and PD-1/PD-L1 in oncology).

Antibody-drug conjugates (ADCs) are targeted cancer therapies that deliver potent cytotoxins and radioactive agents to tumor cells. In another word, ADCs are precision medicine that merge cytotoxicity of small molecules with specificities of mAbs to create potent and specific cancer drugs. Advancement in antibody engineering and technical maturation of site-specific conjugations have resulted in improved efficacy and stability of both payload and antibody [8]. As a result, over 60 ADCs were in clinics, with 11 approved by 2020 (Table 3).

Bispecific antibodies (bsAbs) can simultaneously engage two targets and induce changes in cellular signaling such as cancer metastasis and inflammation [9]. Thus far, only two bsAbs have gained regulatory approvals, Blincyto (blinatumomab) in 2014 for cancer indication and Hemlibra (emicizumab) in 2017 for treatment of hemophilia A. Blinatumomab is used to treat B-cell acute lymphoblastic leukemia (ALL) and targets CD19 and CD13 expressed on tumors and T-cells, respectively. Currently there are over 85 bsAbs in clinical development for a wide variety of indications [9]. Examples of investigational bsAbs for anti-cancer immunotherapy include CD28/PSMA bsAb for prostate cancer from Regeneron, CD3/CLEC12A bsAb for liquid tumor from Merus N.V., programmed cell death protein 1 (PD-1)/programmed death-ligand 1 (PD-L1) bsAbs from Eli Lilly and Innovent Biologics for advanced solid tumors, and EGFR/c-Met bsAb from Shanghai EpimAb Biotherapeutics for advanced solid tumors. Multifunctional molecular structures including bivalent monospecific, bivalent bispecific (with albumin conjugates), trivalent bispecific, and bispecific chimeric antigen receptor (CAR) T cell have been developed and entered the clinic [10]. In addition to IgG-based bsAbs, IgM has also been used to create novel T-cell redirected bsAb. IGM-2323, currently in Phase I study, contains ten CD20 and one CD3 [single-chain variable fragment (scFv)] binding arms. IGM-2323 is under investigation for treatment of B cell non-Hodgkin’s lymphoma (NHL) and other B cell malignancies. In vitro studies have suggested a lower cytokine release profile with IGM-2323 compared to an IgG based CD20/CD3 bsAb with the same binding units [11].

The two main classes of therapeutics, antibodies and small molecules, are separated by a huge gap in their molecular weight. Peptides, defined by FDA as polymers containing 40 or less amino acids, can bridge this gap. Peptides are unique and can combine the advantages of small molecules with that of antibodies. As of September 2020, about 67 peptide-based drugs were approved in the US [12]. The two peptide-based drugs (Lutathera and Ga 68 DOTATOC) approved for oncology indication in the past five years are radioactive diagnostic agents for neuroendocrine tumors.

### 1.3. Cell and Gene Therapies

Cell and gene therapies (oligonucleotides, viral vector delivery and gene editing) have recently been given a lot of attention. It is important to note that oligonucleotide-based therapeutics are categorized as novel drugs and are regulated by the Center for Drug Evaluation and Research (CDER) of FDA, whereas cellular and gene therapy products (viral vector based and gene editing therapy) are categorized as ‘Vaccine, Blood and Biologics’ and are regulated by the Center for Biologics Evaluation and Research (CBER) of FDA. The approved cell and gene therapies by CBER are summarized in Table 4.

Cell therapies are genetically engineered live cell products to repair or replace damage cells or tissues. This approach demands extensive characterization to ensure safety, efficacy, compatibility profiles, and lack of off-target activities. Stem and immune cell transplantation qualify as cell therapy. Immune cells can be proliferated and/or genetically engineered to enhance their anti-tumor capability prior to transplant. Different strategies including stem cell, tumor-infiltrating lymphocyte (TIL), engineered T cell receptor (TCR), chimeric antigen receptor (CAR) T cell, natural killer (NK) cell, and macrophage-based therapy have been employed to increase effectiveness of cell therapy. Over 1400 active agents are included in the cancer cell therapy pipeline globally. CD19 and B cell maturation antigen (BCMA) continue to be the highest priority targets in cell therapy against blood cancer in 2020 [13]. Up to date, four cell-based therapies, all for cancer, have been approved. These include a dendritic cell-based cancer vaccine (sipuleucel-T) for prostate cancer and three CAR T therapies for the treatment of non-solid tumors. The most recent CAR T therapy approved by FDA on July 2020, Tecartus (brexucabtagene autoleucel), is for the treatment of relapsed or refractory mantle cell lymphoma (MCL). Almost 87% of patients responded to a single infusion of Tecartus and 62% showed complete response [14]. This remarkable efficacy gained Tecartus a breakthrough designation. Currently, over 600 CAR Ts are being evaluated in the early stages of clinical development as single agent or in combination with other therapeutics for oncology. About 23 active CAR T studies have entered phase III with focus on non-solid tumors, such as multiple myeloma and lymphoma.

Gene therapy is defined as genetic engineering through the use of viral vectors. Currently there are two adeno-associated virus (AAV)-based gene therapies approved by FDA (Table 4). In 2015, FDA approved Imlygic (talimogene laherparepvec) as the first oncolytic virus therapy for melanoma. Oncolytic viruses are genetically modified virus that selectively replicate within tumor cell to induce lysis [15,16]. Imlygic is engineered to express human granulocyte-macrophage colony stimulating factor (GM-CSF). Other oncolytic virus that have entered clinical trials include engineered adenovirus, vaccinia virus, herpesvirus, reovirus, Seneca valley virus, and coxsackievirus [17,18].

Oligonucleotide (ON) therapy blocks translation of disease-causing proteins, including antisense oligonucleotide (ASO), aptamer, short interfering RNA (siRNA), mRNA, ribozyme, and modified mRNA (modRNA) [19]. They can directly target RNA moieties, ribosomes, or proteins in the cytosol [4,19]. Thus far, ten oligonucleotide-based drugs have gained FDA approval, including ASO, aptamer, and siRNA. Cellular uptake and cytosolic delivery of ONs have proved to be a challenging task due their large molecular weight and negative charge. To overcome this challenge, chemical modifications (phosphorothioate linkage and 2′-*O*-methyl replacement) and systemic delivery (lipid nanoparticles and liposome) have been exploited [19]. For example, appending N-acetylgalactosamine (GalNac) to one end of ASO and siRNA strands have shown promise in sustained target knockdown in liver hepatocytes in vivo, confirming successful cellular delivery and <1% endosomal escape [20,21]. There are more than 15 GalNac-conjugated ONs at various stages of clinical development. Givlaari (givosiran sodium) is a GalNac-siRNA conjugate available in the market for the treatment of acute hepatic porphyria in adults [4]. Thus far, there is no approved ON for cancer. To improve ON cellular uptake, conjugation to various carriers such as small molecules, antibodies, peptides, and carbohydrate have been investigated [19,22].

Gene therapy has gained a great deal of enthusiasm after the advent of site-specific genetic engineering called gene editing. Current approaches include Zinc finger nuclease (ZFN), Transcription activator-like effector nucleases (TALENs) and clustered regularly interspaced short palindromic repeats (CRISPR) for DNA targeting [23,24], and adenosine deaminase acting on RNA (ADAR) for site-directed RNA-editing [25,26]. RNA editing is yet to be tested in human and it might offer a safer approach due to transient and potentially reversible edits to RNA instead of DNA [27]. The CRISPR-Cas9-guide RNA allows precise and efficient targeting of DNA [28,29]. However, cellular delivery has hindered its progress as delivering Cas9 protein or mRNA and short guide RNA to target cells remains a challenging task. Combination of the CRISPR/Cas9 technology with CAR T platform created a unique opportunity in 2019 to test the technology in ex vivo format in human studies against cancer and blood disorders. For cancer indication, T cells were harvested from two patients (one with multiple myeloma and one with sarcoma) and were subjected to ex vivo gene editing to disable the PD-1 gene. The edited T-cells were infused back into patients. For blood disorder indication, the harvested bone marrow stem cells were edited to express fetal hemoglobin. The goal was to replace cells with defective hemoglobin by the healthy cells [30,31]. Remarkably, none of the two patients with blood disorders had disease relapse for nine months post therapy [32]. Currently, 27 active early stage CRISPR/Cas-based studies are registered in clinicaltrial.gov (accessed on 1 September 2020) with the majority of indications in non-solid tumors and blood disorders [33].

**Figure 1:**
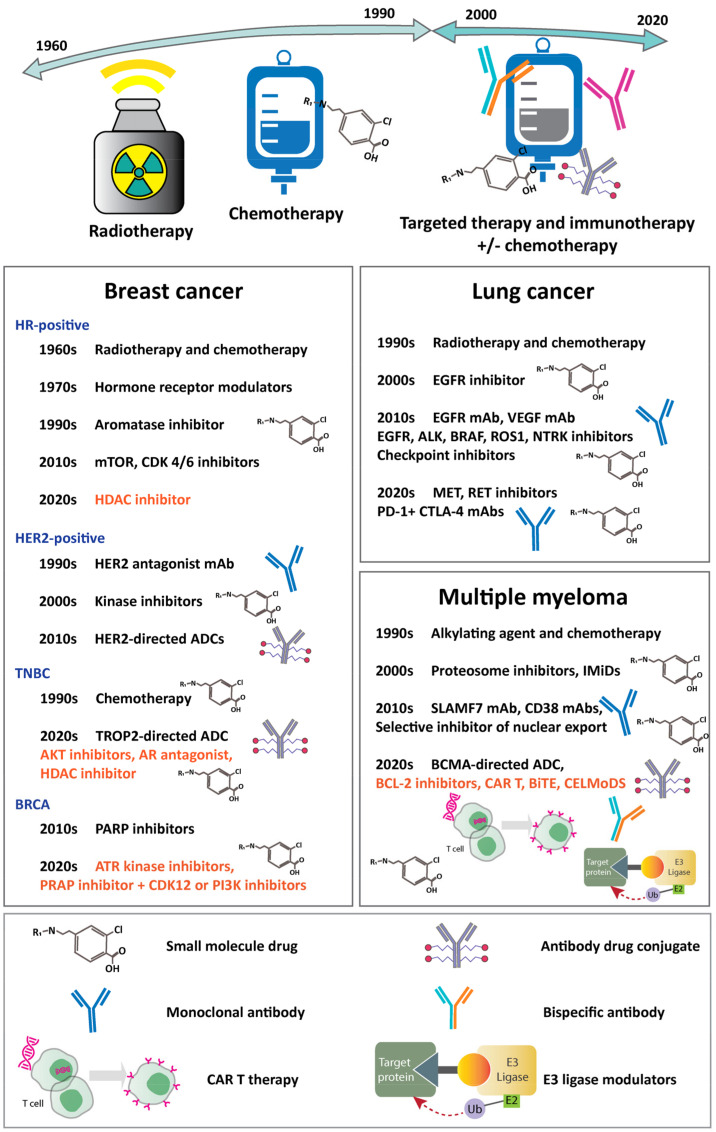
Therapeutic modalities for breast cancer, lung cancer, and multiple myeloma discussed in this article. Approaches shown in black are drugs in the market. Approaches labeled in orange are drugs in clinical development. mTOR: Mammalian target of rapamycin; CDK: Cyclic-dependent kinase; ADC: Antibody-drug conjugate; AKT: Protein kinase B; HDAC: Histone deacetylase; AR: Androgen receptor; PARP: Poly ADP-ribose polymerase; PI3K: Phosphatidylinostitol 3-kinase; EGFR: Epidermal growth factor receptor; VEGF: vascular endothelial growth factor; mAb: Monoclonal antibody; ALK: Anaplastic lymphoma kinase; ROS1: proto-oncogene tyrosine-protein kinase; NTRK: Neutrophilic receptor tyrosine kinase; PD-1: Programmed cell death protein 1; CTLA-4: Cytotoxic T-lymphocyte-associated protein 4; IMiDs: Immunomodulatory drugs; SLAMF7: Signaling lymphocytic activation molecule F7; BCMA: B-cell maturation antigen; BCL-2: B-cell lymphoma 2; CAR T: Chimeric antigen receptor; BiTE: Bispecific T cell engager; CELMoDS: Cereblon E3 ligase modulators.

In this article, we present a comprehensive review of therapeutic modalities in three major types of cancers (Figure 1). We provide an in-depth discussion of the novel therapeutics that are developed in the last five years and summarize the advances and challenges for each approach. The advancement was made possible because of the discovery platform optimization, novel target identification, and innovative technologies. Current trends might help better understand the future developments and inspiration for novel drugs that would benefit patient.

## 2. Breast Cancer

Breast cancer is the most common type of cancer in women. The estimated number of new cases in 2020 is 2.7 million [34]. The stage of breast cancer at diagnosis has remained the most critical factor in determining the survival outcome. However, increased awareness, early detection, and better screening have increased the survival rate in the last three decades. In fact, the death rate in breast cancer patients in the US had declined 40% among women in 2017 compared to 1989 [34]. Half a century ago, breast cancer was considered a local disease and the primary treatment was radical surgery (Mastectomy). Over the time, lumpectomy with radiation has become the primary treatment. FDA approved chemotherapy drugs for breast cancer include taxanes, anthracyclines, and platinum. Compared to the standard schedule of treatment (every three-weeks), combination of doxorubicin, cyclophosphamide, and paclitaxel administered every two weeks with high density has slowed cancer growth and extended survival without introducing additional adverse side effects [35].

Tumor size, involvement of lymph nodes, and receptor expression profile dictate the benefits of chemotherapy in breast cancer patients. A hallmark study in 2001 categorized various breast cancer subtypes and provided a guideline for treatment plans. The four subtypes, Basal-like, HER2/neu-positive, Luminal A, and Luminal B [36] (Table 5), are defined based on the presence or absence of the hormone receptors (estrogen and progesterone receptors) and Human Epidermal Growth Factor Receptor 2 (HER2). In particular, it was shown that activities of 21 genes can influence cancer growth and its response to treatment [37]. The 21-gene oncotype DX recurrence score has been used to sort early-stage ER-positive/HER2-negative breast cancer into a low, medium, or high-risk group. Among the subtypes listed in Table 5, only the high-risk group ER-positive/HER2-negative has benefited from adjuvant chemotherapy. Triple negative breast cancer (TNBC) and HER2-positive breast cancer tend to be more sensitive to chemotherapy but have increased risk of brain metastases [38].

### 2.1. Hormone Receptor Positive (HR-Positive) Breast Cancer

HR-positive subtype of breast cancer is categorized by slow-growing cancer cells that are fueled by overexpressed hormone estrogen receptor (ER) and progesterone receptor (PR). Hormone or endocrine therapy was the first treatment offered to block hormone receptors and/or to lower hormone levels. The first selective estrogen receptor modulator (SERM) called Novaldex (tamoxifen) was approved in 1977 for both pre- and post-menopausal women to control invasive breast cancer [39]. It was shown that tamoxifen administration for 10 years can lower risks of breast cancer recurrence and prevent contralateral breast cancer in premenopausal patients [40]. Faslodex (fulvestrant) is another agent that is approved for HR-positive postmenopausal women with disease progression that had previously received other anti-estrogen therapy. Fulvestrant therapy results in ER degradation, therefore, halts ER signaling. Fulvestrant was approved in 2017 for advanced HR-positive breast cancer. Another class of drugs for systemic treatment of cancer is aromatase inhibitor (AIs). Aromatase is an enzyme that converts androgen into estrogen. It was shown that AIs block estrogen production in certain tissues (other than ovaries) in postmenopausal women. Currently approved AIs for treatment of HR-positive postmenopausal women with disease progression who had previously received tamoxifen include non-steroidal Femara (letrozole), Arimidex (anastrozole), and steroidal Aromasin (exemestane) [41]. The result of 2015 clinical study SOFT suggested that combination of AIs with ovarian suppressor Zoladex (goserelin) or Lupron (leuprolide) is effective in premenopausal women with HR-positive breast cancer. The US Preventive Services Task Force recently recommended to include AIs and SERMs for breast cancer risk reduction in high-risk women [42]. Despite all the advancements, the intrinsic and acquired resistance to the hormonal therapies limits their success. For instance, metastatic breast cancer that has reoccurred post adjuvant therapy can develop resistance to anti-estrogen therapies. Different mechanisms for hormonal resistance include loss of ER in the cancer cells, ER mutation, HER2 mutation, epigenetic alteration, and most importantly Phosphoinositide 3-kinase (PI3K)/AKT/mammalian target of rapamycin (mTOR) mTOR signaling pathway activation [43].

**Figure 2:**
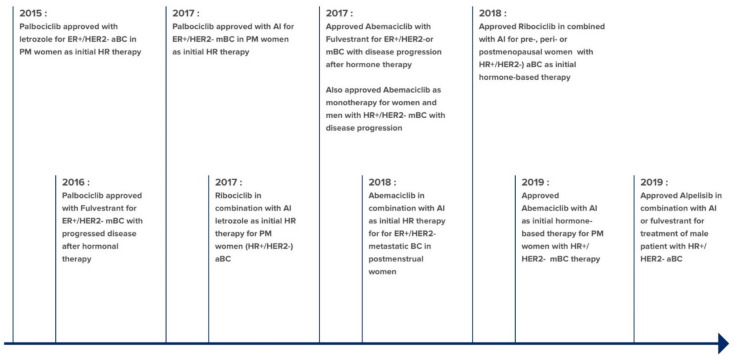
Timeline for the discovery of CDK4/6 inhibitors for HR-positive/HER2-negative breast cancer. aBC: Advanced breast cancer; mBC: Metastatic breast cancer; PM: Postmenopausal.

In the last decade, target specific therapeutics have been developed to address the drug resistance of HR-positive breast cancer. These include drugs against intracellular targets such as mTOR, cyclic-dependent kinase (CDK) 4/6, and *PIK3CA* gene mutation. The mTOR inhibitor Afinitor (everolimus) was approved in 2012 for metastatic HR-positive/HER-negative breast cancer in combination with an AI exemestane in patients whose cancer had progressed despite treatment with letrozole or anastrozole [44]. In 2015, CDK 4/6 inhibitors that block phosphorylation of retinoblastoma protein Rb was approved. Inhibition of Rb phosphorylation leads to cell cycle arrest and reverses endocrine resistance. The approved CDKs inhibitors, Ibrance (palbociclib), Kisqali (ribociclib), and Verzenio (abemaciclib) are to be used in combination with AI as initial hormone-based therapy in post-menopausal women or with fulvestrant in patients whose disease progressed even with hormonal therapy [45]. The timeline of approved CDK 4/6 inhibitors is summarized in Figure 2. In 2019, FDA approved Piqray (alpelisib) in combination with fulvestrant to treat postmenopausal women and men with advanced HR-positive/HER2-negative breast cancer with a *PIK3CA* gene mutation that had occurred during or after treatment with AIs. Randomized SOLAR-I trial estimated improved progression-free survival (PFS) by 11 months with remarkable consistency [46].

Two novel therapies for endocrine resistance metastatic breast cancer include Capivasertib (AZD5363) in combination with fulvestrant and Histone deacetylase (HDAC) inhibitor in combination with exemestane. Capivasertib inhibits PI3K/AKT (E17K), one of the most frequently activated pathways in cancer. Phase II FAKTION trial with capivasertib and fulvestrant showed significantly longer PFS (10.3 months) and improved overall survival (OS) by six months in patients with hormonal therapy-resistant breast cancer [47]. The second novel treatment option is HDAC inhibitor, Epidaza (tucidinostat), in combination with exemestane. HDAC inhibitor is an epigenetic therapy and can reverse the resistance to hormone therapy by increasing histone acetylation. The combination of steroid and AI exemestane is used to target the disease systemically. This combination has shown promising anti-tumor activity in patients with metastatic HR-positive/HER-negative breast cancer with PFS of 7.8 months compared to 3.8 months in placebo group that was given exemestane alone. Patients in this study had previously received hormonal therapy [48].

### 2.2. Human Epidermal Growth Factor Receptor 2/neu Positive (HER2-Positive) Breast Cancer

**Figure 3:**
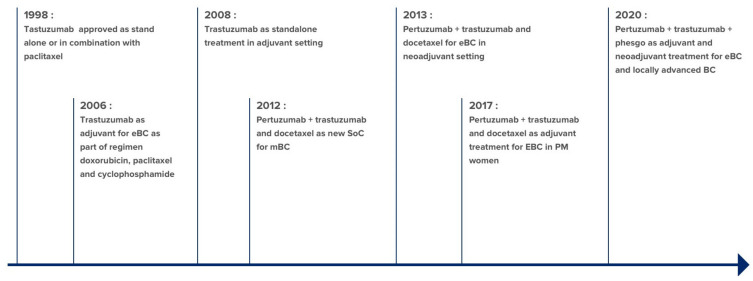
Approval timeline of tastuzumab in combination with different drugs for different stages of breast cancer. aBC: Advanced breast cancer; mBC: Metastatic breast cancer; Ebc: Early breast cancer; PM: Postmenopausal.

One in five women with breast cancer have an amplified transcript of ERBB2/neu oncogene and/or overexpression of growth-promoting protein HER2 [49]. Elevated level of HER2 receptor has been correlated with aggressive disease concomitant with high occurrence rate and mortality [49]. The HER2 humanized antagonist mAb Herceptin (trastuzumab) binds to the extracellular domain of HER2 and blocks its signaling. Multiple approval granted for trastuzumab is depicted in Figure 3. Although a successful treatment for breast cancer, intrinsic and acquired resistance post trastuzumab therapy has limited its use. Another HER2 antibody, pertuzumab, was developed as a neoadjuvant along with trastuzumab to reduce cancer reoccurrence [50]. Interestingly, adding pertuzumab did not increase the rate of heart problems, which was the greatest concern with HER2-targeted therapy. In 2020, FDA approved a new fixed-dose combination of pertuzumab, trastuzumab, and hyaluronidase–zzxf (phesgo) with chemotherapy as neoadjuvant therapy. The combination is now used as part of the complete treatment regimen for locally advanced or early-stage HER2-positive cancer and as adjuvant treatment for early-stage HER2-positive breast cancer with high risk of reoccurrence. This therapy is also approved in combination with docetaxel for HER-positive patients who had not received prior anti-HER2 therapy or chemotherapy [51]. Despite major advances for targeted treatment, 30-50% of advanced HER2-positive patients develop CNS metastases [52]. The onset of symptomatic brain disease can be delayed by the administration of HER2 antibodies but their efficacy is hindered possibly due to the inability of the antibody to cross the blood-brain barrier (BBB) [53].

A significant breakthrough in the treatment of HER-positive breast cancer came from the discovery of kinase inhibitors. Kinase inhibitors can penetrate BBB with much higher efficacy compared to antibodies, hence they can be useful against brain metastasis [54]. The first approved tyrosine kinase inhibitor to treat HER2-positive metastatic breast cancer was Tykerb (lapatinib), which was used in combination with capecitabine [52]. The addition of the chemotherapy agent capecitabine strongly enhanced the efficacy of Lapatinib. Nerlynx (neratinib), an irreversible pan-HER tyrosine kinase inhibitor was the first extended adjuvant therapy approved in 2017 [50]. Three years later, Neratinib was approved in combination with capecitabine for treatment of advanced or metastatic HER2-positive breast cancer in patients who had received two or more prior anti-HER2 therapies. Combination therapy with Neratinib and capecitabine showed 24% reduction in disease progression or death compared to Lapatinib plus capecitabine. The reported PFS for Neratinib with capecitabine and Lapatinib with capecitabine were 47% vs. 38% at 6 months, 29% vs. 15% at 12 months, and 16% vs. 7% at 18 months, respectively. More importantly, treatment with Neratinib and capecitabine significantly delayed the time of intervention for symptomatic disease in CNS. The intervention therapy mostly included ionizing radiation treatment, which was needed less for neratinib arm (11% vs. 15%) [51]. Another HER2 selective inhibitor, Tukysa (tucatinib) was approved in 2020 in combination with trastuzumab and capecitabine for patients with metastatic unresectable or advanced HER2 breast cancer with brain metastases who had received prior anti-HER2 therapy. HER2CLIMB trial showed improved PFS, OS, and overall response rate (ORR) in patients who received combined modalities compared to the patients that received trastuzumab and capecitabine. This newly approved combination with the addition of tucatinib decreased the risk of death by 37%. More importantly, the disease progression or death was reduced to 52% in patients with brain metastases [55]. Disease prognosis was compared in recurrent and/or metastatic ERBB2-positive breast cancer patients treated with neratinib plus paclitaxel vs. trastuzumab plus paclitaxel as the first line of treatment. It was shown that the incidence of symptomatic or progressive CNS reoccurrence was lower (10.1 months vs. 20.2 months) with neratinib plus paclitaxel and the timeline of CNS metastases was delayed. While promising, this result needs to be confirmed in a larger clinical study [56]. In another trial (TBCRC 022), patients with progressive HER2-positive brain metastases received neratinib along with capecitabine. Two additional cohorts of patients, Lapatinib naïve (cohort 3A) and Lapatinib treated (cohort 3B), were also enrolled in this trial. The composite CNS ORR was 49% in cohort 3A vs. 33% in cohort 3B. Median PFS was 5.5 and 3.1 months and median survival was 13.3 and 15.1 months in cohorts 3A and 3B, respectively [57]. Overall, tyrosine kinases have offered promising results when used in combination with other modalities for treatment of HER-positive breast cancer.

Another landmark in breast cancer treatment was the introduction of a two-in-one antibody-drug conjugate (ADC). Unlike traditional chemotherapy, ADCs are intended to target cancerous cells. Properties of the two approved ADCs against HER2-positive breast cancer is summarized in Table 6. Kadcyla or T-DM1 (ado-trastuzumab emtansine) was first-in-class ADC approved as monotherapy [58] to treat HER2-positive breast cancer patients with progressed disease despite prior trastuzumab and taxane therapy. T-DM1 is also approved as adjuvant therapy [59] for early breast cancer patients with residual invasive disease after receiving neoadjuvant taxane and trastuzumab. Despite high efficacy and improved treatment outcome, primary and acquired resistance is frequently developed against T-DM1. A variety of explanations is provided for the resistance and downmodulation of HER2 is identified as a common reason [60]. Second-generation ADC, Enhertu or T-Dxd (fam-trastuzumab deruxtecan-nxki), was approved in December 2019 to treat metastatic or unresectable HER2-positive tumors in patients who were previously treated with at least two anti-HER2 drugs. A cleavable linker was designed to promote drug release to extracellular space to cause a stronger bystander effect. In addition, higher drug to antibody ratio (DAR) was implemented in the design of ADC to deliver a higher payload to tumor cells that have lower HER2 expression [61]. Currently, three trials are ongoing to explore the efficacy of T-DXd. These include DESTINY-BREAST02 (T-DXd vs. standard of care T-DM1) [62], DESTINY-BREAST03 (T-DXd vs. T-DM1) [63], and DESTINY-BREAST04 (T-DXd vs. chemotherapy in HER2 low expressing disease) [64] (Table 7).

Currently, there are several HER2 targeting ADCs in different phases of clinical trials [65] (Table 7), including ADCs against HER3 and novel site-specific conjugates. HER3 overexpression in breast cancer (50–70%) is associated with diminished survival, however, no effective treatment is available [66]. U3-1402 is the first-in-class investigational anti-HER3 ADC. In traditional ADCs, surface exposed lysine or cysteine residues are utilized for conjugation of the cytotoxic payload. The non-specific conjugation might result in variability in DAR and conjugation sites, as well as ADCs that are often unstable in systemic circulation. To overcome this limitation, non-natural amino acids were incorporated into the recombinantly expressed antibody. An example is ARX788, in which a cytotoxic payload is conjugated to the non-canonical amino acid (para-acetyl phenylalanine) in the heavy chain at a predetermined site [67].

**Table 7:** ADCs against HER2 breast cancer in clinical trials.

Investigational ADC	Antibody	Cytotoxic Payload	Linkage	DAR	Clinical Results	Remarks	Reference
SYD985	Trastuzumab	vc-*seco*-DUBA (cause DNA alkylation)	Cleavable linker	2.8	Phase I showed efficacy to T-DM1 resistant and low HER2 expressing aBC	Fast-track designation from FDA	[68]
ARX788	Trastuzumab	Amberstatin269 (AS269) (cytotoxic tubulin inhibitor)	Stable oxime bond	2	Phase I showed dose-dependent anti-tumor activity against HER2+ breast xenograft tumors	Novel site-specific conjugation	[69]
U3-1402	Patritumab	Topoisomerase I inhibitor (DXd)	Peptide-based cleavable linker	7-8	Phase I/II ORR 43.9%	HER3 targeting	[70]

vc-*seco*-DUBA: Valine-citrulline-*seco* duocarmycin hydroxybenzamide azaindole. HER2+: HER2-positive; Dxd: Dexatecan derivative.

A clever interplay on antibody mediated immunotherapy led to the development of margetuximab. The Ab binds HER2 on tumor cells while engaging Fc receptors (FcyRs) on immune cells, causing eradication of cancer cells through antibody-dependent cellular cytotoxicity (ADCC). Margetuximab exhibits comparable HER2 binding and antiproliferative effects as trastuzumab. Fc region of the antibody is engineered for improved binding affinity to CD16A to enhance engagement of the immune system, in particular NK cells. Ongoing phase III SOPHIA trials showed margetuximab combined with chemotherapy significantly improves PFS and reduces risk of progression in heavily pretreated patients with metastatic breast cancer compared to trastuzumab plus chemotherapy. The benefits were enhanced in patients with low-affinity CD16A-158F genotype (32% reduction in disease progression), which is associated with a diminished response to trastuzumab [71]. Utomilumab is another antibody-based therapy with similar concept. Utomilumab binds to the checkpoint receptor 4-1BB (CD-137) that is expressed in activated CD4^+^ and CD8^+^ T cells and NK cells to activate immune response against tumor cells.

### 2.3. Triple Negative Breast Cancer (TNBC)

TNBC is a heterogeneous cancer that is associated with poor prognosis. Lack of therapeutic targets (ER-, PR- and HER2-) limits treatment options. Approved chemotherapy regimen includes platinum compounds, anthracyclines, and taxanes, which are used in both adjuvant and neoadjuvant setting. Common first-line treatment for TNBC is the combination of anthracycline and taxanes followed by capecitabine upon disease progression [72]. Chemoresistance and high frequency of relapsed disease coupled with metastases are key challenges for treatment of TNBC. Only a small population of patients with early TNBC have shown chemosensitivity, while patients with advanced disease respond poorly to standard chemotherapy regimen [73].

TNBC can be further divided into six subtypes based on the tumor gene expression profiles. The six subtypes include two basal-like (BL1 and BL2), mesenchymal (M), mesenchymal stem-like (MSL), immunomodulatory (IM), and luminal androgen receptor (LAR) [74]. Each subtype has unique biological features and deregulation signature of specific signaling pathways [75]. The subtype BL1 is characterized by its high proliferative nature. BL2 subtype is characterized by growth factor signaling pathways, glycolysis, and gluconeogenesis. Both the mesenchymal subtypes M and MSL subtypes are heavily associated with increased expression of genes involved in cell motility, cellular differentiation, and cell growth. IM subtype is identified by factors involved in immune processes and cell signaling. The LAR subtype is characterized by hormonal regulated pathways and dependence on androgen receptors [75].

The molecular subtype profiling is used as a guideline to determine what therapies are best suited for certain patient population. Researchers have investigated intracellular signaling pathways such as PI3K/AKT/mTOR (PAM), poly ADP-ribose polymerase (PARP), androgen receptor network, and cancer driven genes like *BRCA* and *PIK3CA* to develop new therapeutics. The PI3K/AKT1/mTOR pathway is responsible for multiple cellular processes such as cell survival, metabolism, proliferation, and angiogenesis [76]. This signaling pathway is frequently triggered in TNBC due to activating mutations in *PIK3CA* or *AKT1* and/or inactivating mutation in *PTEN* [76]. The PIK3CA activating mutations appear to be more prevalent in mesenchymal and LAR molecular subtypes. The deficient expression of PTEN is common in TNBC and is shown to be associated with activation of AKT pathway, poor prognosis, and resistance leading to tumor growth and survival [77]. AKT inhibitors, ipatasertib and capivasertib, have shown promising results in phase II trials. Inhibition of PI3K/AKT pathway by ipatasertib might even contribute to the reversal of T-cell-mediated immunotherapy resistance [78,79]. The two clinical trials LOTUS and PAKT indicate that the addition of AKT inhibitors to the first-line paclitaxel therapy for TNBC improves PFS, although benefits were limited mostly to *PIK3CA*/*AKT1*/*PTEN* altered tumors. These finding identifies PI3K/AKT signaling cascade as a promising therapeutic target of TNBC. A few promising novel targets in clinical development for treatment of TNBC are summarized in Table 8.

Androgen receptor (AR) is expressed in 10–15% of TNBC patients. LAR subtype is characterized with enriched AR expression, making it a potential therapeutic target for this subtype. The AR antagonists including enzalutamide [85], Casodex (bicalutamide) [86], and Seviteronel (VT-464) [87] alone or in combination with different modalities are currently under investigations.

Elevated level of lymphocytic infiltration in TNBC patients suggests higher immunogenicity and potentially higher responsiveness to immunotherapies [88]. In some cases, adding chemotherapy to the treatment regimen was shown to recruit T-cells to the tumor microenvironment [89]. In 2019, Tecentriq (atezolizumab), a PD-L1 checkpoint inhibitor in combination with Abraxane (albumin-bound paclitaxel) was approved for unresectable locally advanced or advanced TNBC. The approval was based on the phase III Impassion130 trial, in which PFS was improved by nearly three months [90]. Administration of PD-1 inhibitor, Keytruda (pembrolizumab) in combination with chemotherapy vs. placebo and chemotherapy in untreated patient population with locally recurrent inoperable or metastatic TNBC also showed promising results in PSF and pathological complete response (pCR) [91]. Other immune checkpoint inhibitors that have been explored for treatment of TNBC include LAG3 and TIM3 antibodies [92].

Trodelvy (sacituzumab govitecan) was the first novel ADC approved in 2020 for relapsed, refractory, or metastatic TNBC patients who had received at least two prior therapies. The mAb component of Trodelvy, sacituzumab, is against pan-epithelial cancer antigen called Trop2 that is expressed in more than 90% of TNBC cells. Sacituzumab is conjugated to an anti-neoplastic topoisomerase I inhibitor called SN-38, which interrupts the DNA replication in cancer cells. About 55.6% of patients that responded to Trodelvy maintained their response for six or more months [93]. SGN-LIV1A (ladiratuzumab vedotin) is another ADC comprised of a humanized antibody against zinc transporter LIV-1 conjugated to an anti-tubulin drug called monomethyl auristatin E (MMAE) via a protease-cleavable linker. SGN-LIV1A is currently in phase I clinic alone or in combination with trastuzumab [94]. Additionally, efficacy of SGN-LIV1A in combination with pembrolizumab is being evaluated as first-line therapy in patients with unresectable locally-advanced or metastatic TNBC [95].

Fibroblast Growth Factor Receptors (FGFRs) are over-expressed in TNBC cells. FGFRs are a family of four highly conserved transmembrane receptor tyrosine kinases (RTK) namely FGFR1, FGFR2, FGFR3, and FGFR4. Activation of FGFR stimulates cell growth, survival, and differentiation. Around 9% and 4% of TNBC cells have amplified FGFR1 and FGFR2, respectively, making FGFRs potential targets for basal-like TNBCs. Two multi-tyrosine kinase inhibitors currently in clinic, Dovitinib and Erdafitinib, have shown FGFR inhibitory activity [96].

Panobinostat is a potent HDAC inhibitor under investigation for metastatic TNBC. Additional HDAC inhibitors tested in combination with chemotherapy or immune checkpoint inhibitors are romidepsin [97] and entinostat [98]. Other experimental targets under investigation for TNBC includes AMP-activated protein kinase (AMPK), Mouse Double Minute 2 Homolog (MDM2), E3 ubiquitin-protein ligase, Metadherin (MTDH), and cell cycle regulating targets such as Aurora kinase, ATR, CHK1, WEE1, CDC25, and Heat Shock Protein 90 (HSP90) [99].

#### BRCA (BReast CAncer Gene) Mutation

The lifetime risk of developing breast cancer in individuals that carry *BRCA1* and *BRCA2* mutation is 72% and 68%, respectively, compared to 12% in noncarriers [100]. BRCA1 and BRCA2 are considered as tumor suppressors responsible for the repair of dsDNA via homologous recombination repair (HRR) pathway. RAD51 assay is used to identify patients with increased risk of homologous recombination deficiency (HRD) and, therefore, can be utilized to identify patients with BRCA mutation [101]. Mutations in BRCA cause dysfunction of the HRR pathway, preventing DNA repair. As a result, cancer ensues. Since a significant amount of DNA damage leads to cell death [102], pathways such as PARP1 are exploited to treat patients. PARP1 (Poly ADP-ribose polymerase) repairs single-stranded DNAs. PARP1 inhibitors halt the enzyme, preventing repair of single-stranded DNA breaks. Single-stranded breaks will translate into double stranded breaks that cannot be repaired by HRP pathway due to BRCA mutation. Consequently, cancer cells die [103]. Currently approved PARP inhibitors are Lynparza (olaparib) and Talzenna (talazoparib). Olaparib is the first-in-class PARP inhibitor approved in 2018 for metastatic breast cancer with germline mutation (gBRCA) but not for patients with a somatic mutation or low expression of BRCA [104]. FDA-approved genetic test (BRACAnalysis CDx) is used to identify patient population eligible to receive olaparib. Less severe AEs were observed with olaparib (37 vs. 50%) compared to the chemotherapy-treated group [104]. Several other large clinical trials are underway to investigate olaparib in somatic mutation in combination with radiation, chemotherapy, and immunotherapy [105]. Another PARP inhibitor talazoparib was approved in 2019 for metastatic breast cancer with gBRCA mutation in patients who had received no more than three prior cytotoxic chemotherapy regimens. PFS and ORR were improved in talazoparib arm compared to the group receiving standard chemotherapy. Main reported AE included hematologic grade 3–4 and non-hematologic grade 3 in talazoparib arm [106].

Unfortunately, patients develop resistance to PARP inhibitors through homologous recombination (HR) by *BRCA* undergoing a second alteration to repair itself [107]. If HR is prevented, tumor cells will remain HR deficient and can be treated with PARP inhibitors. This would be useful for patients who were initially sensitive to PARP and the ones with normal BRCA protein if cancer cells are sensitized to PARP. The ongoing clinical trials to sensitize HR-proficient cancer cells include combination of PARP inhibitors with CDK12 inhibitor (dinaciclib) to block phosphorylation of BRCA1 [108].

Alternatively, BRCA mutation can be treated with sapacitabine that acts on HR pathway [109]. Combination therapy of sapacitabine and olaparib (PARP inhibitor) is investigated in phase II trial [110]. Ataxia telangiectasia mutated protein (ATM) deficiency predisposes cells to become cancerous and it is noted that expression of both ataxia telangiectasia and Rad3-related (ATR) kinase are required for cell survival [111]. A novel ATR kinase inhibitor, BAY 1895344, has shown strong efficacy in cancer xenograft models deficient in DNA damage repair (DDR) as monotherapy or in combination with DDR inhibitors or PARP inhibitors. Additionally, BAY 1895344 in combination with nonsteroidal androgen receptor antagonist darolutamide and eternal beam radiotherapy (EBRT) resulted in improved antitumor efficacy [112]. Future directions in TNBC include combination therapies using multiple agents and diagnosis at earlier-stage.

In the last few decades, FDA has approved more drugs for breast cancer than for any other solid tumors. The novel therapeutics in the field include kinase inhibitors, ADCs, and immunotherapies. These advancements hold promise for addressing the unmet needs for patients, especially for patients with metastatic breast cancer and TNBC.

## 3. Lung Cancer

Lung cancer is the leading cause of death among cancer-related mortalities worldwide. It is estimated to result in 2.2 million new cases and 1.3 million deaths in 2020 [113]. Lung cancer is a disease of the elderly (>60 years) and it can remain asymptomatic until the advanced stages [114]. Early detection using low dose computed tomography (LDCT) has led to a 4–20% reduction in mortality among the high-risk population. However, use of LDCT introduces the risk of exposure to radiation, requires follow-up tests such as biopsy and FDG-PET to confirm the result, and most importantly, it does not detect all types of cancer. Lung cancer is categorized into non-small cell lung cancer (NSCLC) and Small cell lung cancer (SCLC). NSCLC comprises 85% of lung cancers, including adenocarcinoma, squamous, large cell and bronchial carcinoid. SCLC is strongly associated with smoking, it is more aggressive, and grows rapidly [115].

Twenty years ago, the conventional treatment regimen for lung cancer included surgery, chemotherapy, and radiation therapy. Patients with early-stage, localized NSCLC (Stage 1 and 2) were successfully treated by surgery. About 40% of NSCLC patients were diagnosed with lung cancer at stage IV [114]. For this population, adjuvant chemotherapy and common radiation therapy (extreme radiation therapy and brachytherapy) were used [116]. For SCLC, surgery was rarely practiced because around 70% of SCLC patients were diagnosed with extended disease [114]. Although patients often relapse, SCLC is very sensitive to chemotherapy and radiotherapy. Topoisomerase-I inhibitor, Hycamtin (topotecan), approved in 1996 remains the most reliable chemotherapeutic agent to manage relapsed SCLC. In 2007, oral Topotecan was approved [117]. The most common standard of care for SCLC includes chemo drugs Etopophos (etoposide) or Camptosar (irinotecan) plus a platinum-based drug [118].

The use of conventional therapies is limited due to patient old age and side effects caused by lack of specificity. This drawback was overcome by targeting the tumor promoting tyrosine kinases that were identified by genomic testing for personalized treatment. The currently approved therapies for lung tumors with genetic variation are summarized in Table 9 [119]. Most of the tyrosine kinase blockers are either small molecule inhibitors or antibodies that block tyrosine kinase receptors on cancer cells. In 2016, FDA approved the liquid biopsy test (Cobas EGFR mutation V2) for NSCLC patients. This test detects 42 EGFR mutations, including exon 19 deletion, T790, and L858R mutations [120].

Currently, molecular targeted therapy is being investigated for SCLC in the clinic [121]. Aurora A kinase, a key regulator of mitosis is a potential target due to its high expression in SCLC. Takeda’s aurora A kinase inhibitor Alisertib is in phase II with promising results of improved PFS [122]. Unfortunately, resistance to tyrosine kinase inhibitors remains a challenge for patients.

**Table 9:** Approved therapies for NSCLC patients with genetic aberration.

Target Gene and Frequency in NSCLC	Mutation	Targeted Drug	Molecule Type	Drug Generation	Approved Year	Reference
EGFR (20%)	EGFR exon 19 deletion (del19) or exon 21 (L858R) substitution	Tarceva (erlotinib), Iressa (gefitinib)	SM	1st	2004; 2004	[123]
		Gilotrif (afatinib) or Vizimpro (dacomitinib)	SM	2nd	2013; 2018	
	T790 mutation	Tagrisso (osimertinib)	SM	3rd	2018	
EGFR ECD		Portrazza (necitumumab)	Ab	1st	2015	
ALK (5%)	Gene fusion with EML4	Xalkori (crizotinib)	SM	1st	2011	
	L1169M and C1156Y mutations	Zykadia (ceritinib), Alecensa (alectinib), and Alunbrig (brigatinib)	SM	2nd	2017	
	G1202R mutation	Lorbrena (lorlatinib)	SM	3rd	2018	
BRA-F (1–2%)	V600E mutation	Tafinlar (dabrafenib) with Mekinist (trametinib) which is a MEK inhibitor	SM	1st	2017	
ROS1 * (1–2%)	Genetic translocation	Xalkori (crizotinib), Zykadia (ceritinib), Alecensa (alectinib)	SM	1st	2017	
		Rozlytrek (entrectinib)	SM	1st	2019	
VEGF	Angiogenesis Inhibitor	Avastin (bevacizumab), Cyramza (ramucirumab) both combined with chemotherapy	Ab			
NTRK (>1%)	Mutation	Rozlytrek (entrectinib)	SM	1st	2019	
		Vitrakvi (larotrectinib)		1st	2018	
MET (4%)	MET exon 14 skipping deletions	Tabrecta (capmatinib)	SM	1st	2020	[124]
RET (1–2%)	Gene fusion and mutation	Retevmo (selpercatinib) Gavreto (pralsetinib) [125]	SM	1st	2020	[126]

EGFR: Epidermal growth factor receptor; ECD: Extracellular domain; ALK: Anaplastic lymphoma kinase; ROS1: Proto-oncogene tyrosine-protein kinase; EML4: Echinoderm microtubule-associated protein-like 4; VEGF: Vascular endothelial growth factor; NTRK: Neutrophilic receptor tyrosine kinase; SM: Small molecule; Ab: Antibody; ROS1 * shows a high degree of structural homology with ALK.

**Figure 4:**
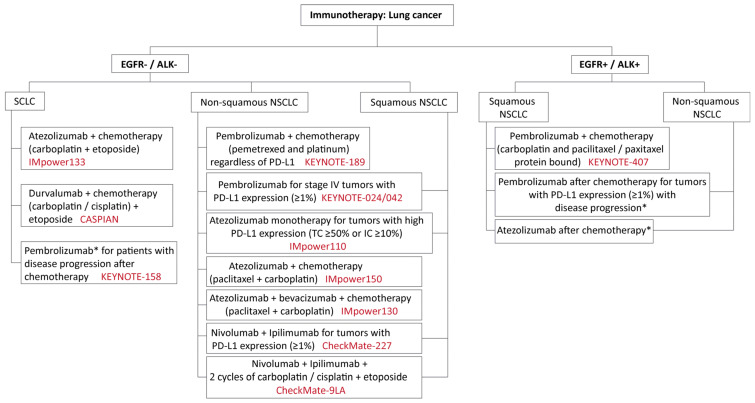
Approved Immunotherapies for Lung cancer. PD-1: Programmed death protein-1; PD-L1: Programmed death ligand-1; NSCLC: Non-small cell lung cancer; SCLC: Small cell lung cancer; TC: Tumor cells; IC: Immune cells. * Either second-line or third-line treatment. Highlighted in red are the clinical trial names.

Most drugs for lung cancer were small molecule inhibitors until 2015, when advent of immunotherapy created a paradigm shift in the treatment of lung cancer. Immunotherapy targets immune checkpoint inhibitor (ICI) proteins to help restore T-cell response to fight cancer. Examples include antibodies against PD-1 [Opdivo (nivolumab) and Keytruda (pembrolizumab)], PD-L1 [Tecentriq (atezolizumab), Imfinzi (durvalumab)], and CTLA-4 [Yervoy (ipilimumab)]. The approved immunotherapies, as monotherapy or in combination with chemotherapy, for lung cancer are summarized in Figure 4. The first dual immunotherapy, PD-1 inhibitor (nivolumab) plus CTLA-4 inhibitor (ipilimumab) was approved in 2020 [127,128]. We have summarized the efficacy data of the first-line immunotherapies for EGFR^-^/ALK^-^ in Table 10. Tyrosine kinase inhibitors are used as the first line of treatment for EGFR^+^/ALK^+^ lung cancer.

In general, the unresectable NSCLC at stage III/IV have a poor prognosis with chemoradiotherapy being the only treatment option. However, PD-L1 inhibitor, durvalumab given as consolidation therapy, has generated hope for the patients. Efficacy of durvalumab was investigated in the locally advanced unresectable stage III NSCLC in PACIFIC trials. The antibody showed remarkable improvement in PFS by 11.2 months. The adverse effect including pneumonia, rash, and diarrhea were more common in patients on durvalumab therapy vs. placebo. In 2019, the three-year OS for durvalumab was 57% compared to 43.5% in placebo. FDA granted durvalumab a breakthrough therapy designation in 2017 [129].

**Table 10:** Efficacy data of immunotherapies approved as first-line of treatment for lung cancer.

Type of Lung Cancer	Trail Name	Combination	OS (months)	PFS (months)	ORR (%)	Reference
Non-squamous NSCLC	KEYNOTE-189	Pembrolizumab + chemotherapy	NR	8.8	48	[130]
		Chemotherapy alone	11.3	4.9	19	
	IMpower150	Atezolizumab + bevacizumab + chemotherapy (paclitaxel + carboplatin)	19.2	8.3	64	[131]
		Bevacizumab + chemotherapy (paclitaxel +carboplatin)	14.7	6.8	48	
	IMpower130	Atezolizumab + chemotherapy (paclitaxel +carboplatin)	18.6	7	49	[132]
		Chemotherapy (paclitaxel +carboplatin)	13.9	5.5	32	
Non-squamous NSCLC and Squamous NSCLC	KEYNOTE-024	Pembrolizumab only	30	10.3	45	[133]
		Chemotherapy	14.2	6	28	
	CheckMate-227	Nivolumab + Ipilimumab	17.1	5.1	36	[127]
		Chemotherapy (carboplatin/cisplatin)	14.9	5.6	30	
	CheckMate 9LA	Nivolumab + Ipilimumab + carboplatin/cisplatin	14.1		38	[128]
		Ipilimumab + carboplatin/cisplatin	10.7		35	
	IMpower110	Atezolizumab only	20.2	8.1	38	[134]
		Chemotherapy (cisplatin or carboplatin)	13.1	5	29	
SCLC	IMpower133	Atezolizumab + chemotherapy (carboplatin + etoposide)	12.3	5.2		[135]
		Chemotherapy (carboplatin + etoposide)	10.3	4.3		
	CASPIAN	Durvalumab + chemotherapy (carboplatin/cisplatin) + etoposide	13	5.1	68	[136]
		Chemotherapy (carboplatin/cisplatin) + etoposide	10.3	5.4	58	

ZEPZELCA (lurbinectedin), a potential new treatment for relapsed SCLC since topotecan’s approval in 1996, was granted accelerated approval in 2020. This selective oncogenic transcription inhibitor is approved as second-line therapy for metastatic SCLC with disease progression during or after platinum-based chemotherapy. Small molecule lurbinectedin is an analog of ET-736 found in sea squirt *Ecteinacidia turbinate*. Clinical studies of lurbinectedin demonstrated 35% ORR and 4.6 months PFS in patients with a chemotherapy-free interval of ≥90 days [137]. In the same year, a first-in-class therapy was approved for patients with *MET and RET* gene alteration. *MET* exon 14 skipping mutation is present in 3–4% of NSCLC and is associated with poor prognosis [138]. Kinase inhibitor Tabrecta (capmatinib) is the first FDA approved therapy for treatment of NSCLC with *MET* exon 14 skipping mutation. The trial demonstrated ORR of 68% (with 4% complete response and 64% partial response) in naïve patients and 41% partial response in previously treated patients [124]. *RET* encodes single pass transmembrane receptor tyrosine kinase. *RET* fusions or rearrangements are somatic juxtapositions of 5′ sequences of other genes with 3′ *RET* sequence. Two selective oral *RET*-inhibitor approved for *RET*-fusion positive metastatic NSCLC are Retevmo (selpercatinib) and Gavreto (pralsetinib) [126]. Cyramza (ramucirumab) combined with EGFR inhibitor Tarceva (erlotinib) were approved in June 2020 as the first-line treatment for metastatic NSCLC patients with *EGFR* exon 19 deletions or exon 21 (*L858R*) mutation. Ramucirumab was discovered from phage display library and its use extended PFS when combined with erlotinib vs. placebo plus erlotinib [139]. FDA also approved the diagnostic test called Oncomine Dx target to identify metastatic NSCLC patients with *RET*-fusion eligible for pralsetinib [125].

*EGFR* exon 20 insertion mutation is the third common *EGFR* mutation. Unlike common *EGFR* exon 19 deletion, which comprises 45% of *EGFR* mutations, *EGFR* exon 20 has low frequency of occurrence (5–10%) and is associated with inducing resistance to *EGFR* inhibitors [140] and poor prognosis. Mobocertinib, a small molecule kinase inhibitor, target EGFR with exon 20 activating insertions. The therapy was granted a breakthrough status based on the phase II trial data with 43% ORR [141]. There are ongoing phase II/III trials to check the effect of LCT in combination with tyrosine kinase inhibitors and immune checkpoint blockers. Another ongoing trial called Lung-MAP is an umbrella trial for testing genetic changes in NSCLC [142]. Liquid biopsy of plasma circulating DNA (ctDNA) can provide results faster than a tissue biopsy to identify mutations. It is anticipated that liquid biopsy might be used for monitoring the treatment response after therapies.

High molecular heterogenicity and complexity of lung cancer demands diverse treatment options and early detection techniques. Novel approaches such as molecular targeted therapy and immunotherapy have changed the treatment landscape, however, chemotherapy remains the main therapy. Survival rate for lung cancer patients in the advance stages remains low, emphasizing the necessity for novel modalities for treatment of this cancer.

## 4. Multiple Myeloma

Multiple myeloma (MM) is a heterogeneous cancer of plasma cells originating in the bone marrow and accounting for 10% of all hematologic malignancies [143]. Accumulation of malignant plasma cells in bone marrow results in bone lesions and growth suppression of red blood cells, causing anemia. MM is associated with the production of abnormal antibody (M protein), which can build up in blood and kidney, resulting in renal abnormalities. Two decades ago, treatment regimen for MM was limited to alkylating agents such as Alkeran (melphalan), Cytoxan (cyclophosphamide), and traditional chemotherapy with corticosteroid Decadron (dexamethasone), and Rayos (prednisone) for palliative care. Melphalan plus prednisone remained mainstay therapy for MM [144] until 1990s, when it was observed that administration of high dose Melphan followed by autologous stem cell transplant (ASCT) results in improved response rate (RR). Since then, this approach has remained a standard therapy for MM [145]. Drug resistance and relapse are the main challenges for treatment of MM.

Based on the clinicopathological criteria, hallmark characteristics of MM include the evidence of monoclonal plasma cell proliferation (>10% plasma cells are plasmacytoma) and end-organ damage including hypercalcemia, renal insufficiency, anemia, and bone lesions abbreviated as CRAB [146]. Asymptomatic patients with plasmacytoma either have monoclonal gammopathy of undetermined significance (MGUS) or smoldering multiple myeloma (SMM). Lack of early intervention in patients with MGUS and SMM introduce the risk of disease progression to MM. Cytogenetic abnormalities in MM include primary abnormalities that occur at an early stage during the transition of normal plasma cells to the clonal premalignant cells (MGUS/SMM) and secondary abnormalities that occur during disease progression. These abnormalities, summarized in Table 11, can be detected by FISH (fluorescence in situ hybridization) and SNP (Single nucleotide polymorphism) microarray [147]. Genes that are often mutated in MM are *KRAS*, *NRAS*, *TP53*, *DIS3*, *FAM46C*, *BRAF*, *TRAF3*, *ROBO1*, *CYLD*, *EGR1*, *SP140*, *FAT3*, and *CCND1* [148]. Patients with symptomatic MM have the highest risk of disease progression, followed by SMM (10%/year), and MGUS (1%/year) [149].

In 2014, International Myeloma Working Group (IMWG) revised the staging of MM to encourage early diagnosis before end-organ damage [150]. Accordingly, patients with ≥60% clonal bone marrow plasma cells, serum-free light chain (FLC) ratio ≥ 100 (provided involved FLC level is ≥100 mg/L), and more than one focal lesion detected by magnetic resonance imaging (MRI) have more than 80% probability of progression to MM within two years [151]. The new classification was made possible because of progress in imaging modalities, discovery of new drugs, and identification of biomarkers. For example, detection of early bone defects was facilitated by the advanced imagining techniques, such as computed tomography (CT) and fluoro-deoxyglucose (FDG) positron emission tomography/computed tomographic scans (PET/CT).

**Figure 5:**
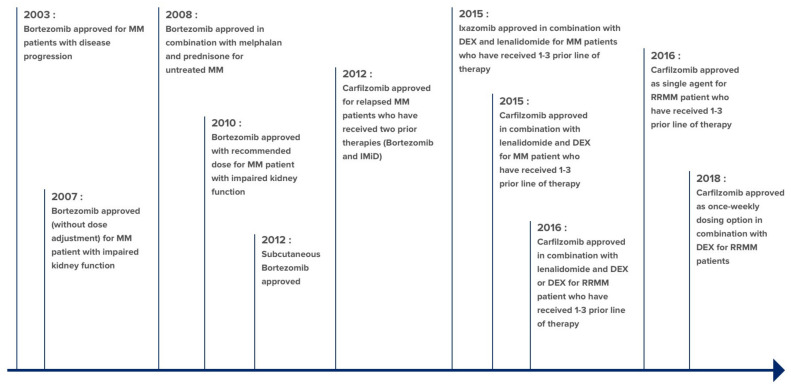
Approval timeline of proteasome inhibitors for multiple myeloma. MM: Multiple myeloma; RRMM: Relapsed/refractory multiple myeloma; DEX: Dexamethasone.

One breakthrough in the treatment of MM was the introduction of first-in-class proteasome inhibitor (PI) Velcade (bortezomib) in 2003. Bortezomib is used as induction therapy for patients eligible or ineligible for ASCT, it is also used as maintenance therapy (MT). Although effective as single agent for relapsed MM, use of bortezomib is limited due to drug resistance and toxicities such as peripheral neuropathy. Intravenously Administered Kyprolis (carfilzomib) is the second-generation PI with increased efficacy and lower toxicity. Shortly after approval of carfilzomib, Ninlaro (ixazomib), the first-in-class oral PI for relapsed refractory MM (RRMM) with improved therapeutic index became available. Notably, bortezomib and ixazomib are borate-based dipeptide drugs that bind reversibly to proteasomes. Carfilzomib is an epoxyketone with irreversible binding mode. The approval history of small molecule PIs for MM is summarized in Figure 5 [152].

Three approved immunomodulatory drugs (IMiDs) for MM were developed by Celgene. In 2006, first-in-class Thalomid (thalidomide) was approved in combination with dexamethasone (DEX) for the treatment of newly diagnosed MM (NDMM) [153]. To enhance potency and reduce the adverse effects of thalidomide, Revlimid (lenalidomide) and Pomalyst (pomalidomide) were approved in 2006 and 2013, respectively. Lenalidomide was originally approved as combination therapy with DEX for patients with NDMM or RRMM who had received at least one prior therapy [154]. Currently, lenalidomide is used as a first-line treatment for MM and is the only drug approved as maintenance therapy (MT) following ASCT [155]. Unfortunately, the majority of the patients treated with IMiDs will develop drug resistance due to the mutation or downregulation of Cereblon, the primary target of IMiDs [156]. Pomalidomide is a hybrid structure of thalidomide and lenalidomide. Pomalidomide combined with a low dose of DEX is shown to be effective in relapsed MM patients with resistance to lenalidomide and PIs [153].

Treatment of NDMM patients, eligible or ineligible for ASCT and MT, with combination of PI, steroid, and IMiDs (thalidomide, lenalidomide, or cyclophosphamide) was a groundbreaking step in treatment of MM. These first-line therapies include bortezomib and dexamethasone combined with thalidomide (VTd), or with lenalidomide (VRd) or with cyclophosphamide (VCd) [157]. Typically, 3-6 cycles of induction regimen are recommended to have the maximum response. The triplet regiments improved RR compared to the previously practiced doublet therapy with DEX combined with either thalidomide (Thal-DEX) or lenalidomide (Rev/Dex). Clinical studies point to the superiority of VTd compared to VCd. However, VCd has lower risk of peripheral neuropathy and skin rash and remains an attractive option for patients with renal impairment [158,159]. VRd is shown to be associated with decreased peripheral neuropathy, increased efficacy, and superior RR compared to VTd [160]. In VRd regiment bortezomib is frequently replaced by the next-generation PIs, such as carfilzomib (KRd) or ixazomib (IRd) to increase potency [161]. NDMM patients, not eligible for transplant, are usually treated with a combination of Melphalan and prednisone (MP) plus thalidomide (MPT), bortezomib (MPV), or lenalidomide (MPR). High-risk MM patients who are not eligible for ASCT are commonly treated with VRd or VCd [152].

Farydak (panobinostat) is an oral HDAC inhibitor that is used with bortezomib and DEX to treat RRMM patients that were previously treated with bortezomib and an IMiD [162]. Panobinostat increases acetylation of proteins involved in oncogenic pathways and has synergistic cytotoxicity in MM when combined with bortezomib. Combination of carfilzomib with panobinostat is currently under investigation in clinic for treatment of patients with relapsed disease who were previously treated with triplet regimen [163]. 

Since MM patients usually relapse, the minimal residual disease (MRD) status has become an important prognostic factor. MRD refers to the minimal number of myeloma cells that remain in the patient’s bone marrow post treatment. More sensitive approaches have recently been developed to increase detection sensitivity to threshold at the level of 10^−6^. These detection methods include next-generation flow cytometry and next-generation sequencing [164].

Another breakthrough in treatment of MM was the development of mAbs targeting resistant MM. Empliciti (elotuzumab) is the first-in-class humanized mAb against glycoprotein CS1, also known as signaling lymphocytic activation molecule F7 (SLAMF7). CS1 is expressed in 95% of bone marrow myeloma and NK cells. It mediates adhesion of myeloma cells to bone marrow stromal cells, promoting their growth and survival. Elotuzumab facilitates interaction between NK cells and myeloma cells to induce ADCC [165]. Elotuzumab was granted the Breakthrough status in 2015 when used in combination with lenalidomide and DEX for RRMM patients who had received one to three prior therapies. Alternatively, elotuzumab is used in combination with pomalidomide and DEX for RRMM patients who had at least two prior therapies with PI and lenalidomide [166,167]. Elotuzumab is currently under evaluation for first-line induction therapy for NDMM (transplant eligible or non-eligible). At present, a phase II trial is ongoing to determine RR of elotuzumab in combination with VRd in transplant eligible NDMM. High-risk cytogenetic patients are treated with elotuzumab-VRd as MT. After four cycles of the induction therapy, ORR was 100% with 24% achieving CR [168].

In 2015, FDA also approved another landmark drug Darzalex (daratumumab), an IgG1-κ fully human antibody directed against CD38. Daratumumab induces ADCC, ADCP, and CDC against MM cells present in bone marrow [169]. A list of different trials with daratumumab in combination with various drugs is summarized in Table 12 [170]. FDA granted daratumumab a breakthrough therapy and orphan drug designation. The infusion time for daratumumab is 7, 4.3, and 3.4 h for the first, second, and subsequent infusions, respectively. In 2019, FDA approved spilled dose of daratumumab to allow infusion of the first dose in two days, but the total infusion time remained the same [171,172]. One year later, FDA approved Darzalex Faspro (daratumumab and hyaluronidase-fihj), a new subcutaneous formulation of daratumumab [172]. This fixed-dose formulation can be administered in 3–5 min.

Another CD-38 targeting antibody, Sarclisa (isatuximab-irfc), was approved in March 2020. The chimeric Ab is used in combination with pomalidomide and low dose DEX for the treatment of RRMM patients who had received multiple prior therapies. Besides killing myeloma cells by CDC, ADCC, and ADCP; Isatuximab induces production of reactive oxygen species downstream of lysosomal-associated pathway, leading to cell death [178]. Based on ICRIA-MM trial, this combination therapy demonstrated a significant improvement in PFS (11.5 months vs. 6.5 months) and ORR (60.4% vs. 35.3%) compared to control arm (pomalidomide and DEX) and showed 40% reduction in disease progression or death [179]. Isatuximab is administered in 3.3 h in first infusion and 2.8 h in subsequent infusions. Isatuximab has been tested in ongoing trials as part of induction therapy in transplant eligible or non-eligible NDMM [180]. MOR22 (TJ202), another CD38 antibody, induces ADCC and ADCP and is currently under investigation in phase III trial in combination with lenalidomide and DEX for RRMM [181].

Xpovio (selinexor), a first-in-class oral selective-inhibitor-of-nuclear-export (SINE), was approved in combination with DEX for RRMM patients who had received at least four prior therapies and whose disease was refractory to at least two PIs, two IMiDs, and one anti-CD38 antibody [125]. This novel drug blocks exportin 1 (XPO1) and forces nuclear accumulation and activation of tumor suppressor proteins such as p53, p73, p21, p27, pRb, FOXOs, BRCA1/2, and IκB-α. It also inhibits NF-κB and reduces oncoprotein translation [182]. Selinexor provides a treatment option for delaying disease progression in patients with no available therapy.

Oprozomib and marizomib are two promising PIs currently in the clinical trial. Oprozomib, currently in phase II, is an oral PI that is structurally related to carfilzomib. The drug has shown efficacy in RRMM patients who were refractory to other PIs [183]. Marizomib, an irreversible pan-PI, is in clinical development for RRMM [184]. A new investigational first-in-class anti-cancer peptide-drug conjugate (PDC) called Melphalan flufenamide (melflufen) is also under development. Melflufen is a peptidase-potentiated alkylating agent that is rapidly taken up by aminopeptidase positive myeloma cells due to its high lipophilicity. Once internalized, it is immediately cleaved by peptidases to deliver an entrapped hydrophilic alkylator payload into cancer cells [185]. Recently, FDA granted priority review of melflufen in combination with DEX for patients with MM whose disease were refractory to at least one PI, one immunomodulatory agent (IMiD), and one anti-CD38 mAb. Melflufen combination therapy elicited ORR of 26% in patients with triple refractory disease [186].

Venetoclax is a selective orally bioavailable B-cell lymphoma 2 (BCL-2) inhibitor with promising result for MM patient with t(11;14) genetic alteration. In t(11;14) translocation, upregulation of the cell survival protein BCL-2 results in disease progress. Phase III BELLINI trial showed that the addition of venetoclax to the combination of bortezomib and DEX significantly improves PFS in the intent-to-treat population (22.4 months) compared to placebo plus bortezomib and DEX (11.5 months). The reported ORR, CR, and MDR negativity were 82%, 26%, and 13%, respectively, with ventoclax regimen vs. 68%, 5%, and 1% in the control arm. Although higher risk of death was observed in ventoclax arm (51 pts) vs. control arm (19 pts), subgroup analysis indicated that patients with t(11;14) alteration had no excess death. In fact, patients with high BCL-2 expression showed improved PFS by 12.4 months, indicating that venetoclax in combination with bortezomib and DEX is effective in patients with t(11;14) genetic alteration but might cause harm to others [187]. The CANOVA trial is currently investigating efficacy of venetoclax in combination with bortezomib and DEX in RRMM patients with t(11;14) translocation [188,189].

CD38 mAbs and SLAMF7 therapies have significantly improved the patient prognosis but MRD persists. To overcome this challenge, a novel cell-surface receptor target, BCMA (B-cell maturation antigen), was exploited for targeted therapy. BCMA is unique to plasma cells and is overexpressed on myeloma cells, making it a promising target with limited risk of off-target toxicity [190]. BCMA promotes plasma cell growth and survival by signal transduction through BAFF (B-cell activation factor) and APRIL (a proliferation-inducing ligand) [191]. Upregulation of BCMA also correlates with disease burden and poor prognosis in MM [190]. Currently, multiple BCMA targeting modalities, including ADCs, bsAbs, and CAR T are being explored. GSK’s belantamab mafodotin (GSK2857916) is a humanized BCMA mAb conjugated to microtubule inhibitor monomethyl auristatin-F (MMAF) [192]. GSK2857916 is a first-in-class ADC approved to treat RRMM patients with prior therapies with IMID, PI, and CD38 antibody [193]. Binding of belantamab to BCMA also enhances ADCC and ADCP in myeloma cells. The FDA approval was based on a DREAMM trial with an ORR of 31% [194]. Additional clinical trials are in progress to evaluate belantamab mafodotin efficacy in refractory MM patients [195]. Other promising ADCs targeting BCMA include MEDI2228 by AstraZeneca [196] and CC-99712 by BMS/Sutro Biopharma.

Another pioneering modality for treatment of MM is BiTE (Bispecific T cell engager). BiTE recruits the patient’s own T cells to fight cancer cell. BiTE is comprised of two flexibly-linked scFvs, with one targeting a tumor-associated antigen on myeloma cell (BCMA) and the other targeting CD3 on T cells [190]. Small size of BiTE enhances penetration to tumor microenvironment but causes fast clearance through kidney. AMG420 (BI 836909) is the first BiTE under investigation for treatment of RRMM that targets BCMA and CD3 [197]. Amgen recently designed a half-life extended (HLE) BiTE molecule by introducing Fc domain. Half-life extended (HLE) anti-BMCA BiTE has shown efficacy in mouse xenograft model [198]. Additionally, combination of daratumumab with the bsAb teclistamab (JNJ-64007957) targeting BCMA and CD3 is under investigation in RRMM patients. The result of phase I trial suggested no safety concerns and 78% ORR at the highest weekly dose [199]. Other promising bsAbs under clinical investigation that target both CD3 and BCMA include CC-93269 (Celgene), REGN5458 (Regeneron), and PF-06863135 (Pfizer). There are many bispecific antibodies for MM in clinic or preclinic targeting CD38, CD138, CD19, CD319, GPRC5D, and FcRL5 [200].

CAR T cell therapy is actively being explored for treatment of MM. In CAR T cell therapy, T cells are collected from the patient and engineered ex vivo by inserting an artificial gene to help them recognize and fight cancer cells. In short, a new T cell receptor that binds to tumor-associated antigens is introduced in patient’s T cell. Most of the CAR T cell therapies for MM target BCMA. Examples include bb21217 (Celgene/bluebird bio), JCARH125 (Celgene/Juno Therapeutics), P-BCMA101 (Poseida Therapeutics), bb2121, JNJ-4528/LCAR-B38M (JNJ/Nanjing legend biotech), and ALLO-715 (Allogene). Bb2121 or idecabtagene vicleucel (Ide-cel) targets BCMA using lentiviral transduction. The expressed molecule is comprised of anti-BCMA (scFv-targeting domain for BCMA), a transmembrane domain fused to cytoplasmic signaling domain of a CD3-zeta activation domain and 4-1BB co-stimulatory domain. Phase II KarMMA trial with Ide-cel demonstrated a significant improvement in ORR in patients with RRMM. Transplant of CAR T cells at the target dose range of 150-450 × 10^6^ resulted in the median ORR of 73.4% [201]. Celgene and Bluebird Bio developed next generation anti-BCMA investigational antibody bb21217 based on BB2121 that might have longer durability due to acquired memory. Enrichment of T-cells with a memory like phenotype was achieved with their incubation with PI3 kinase inhibitor (bb007) ex vivo. Data suggested that this strategy might help cells to persist longer *in vivo*, resulting in prolonged remission [202]. JNJ-4528 (LCAR-B38M) is a CAR T developed by Johnson & Johnson and Nanjing Legend Biotech. JNJ4528 is a structurally-differentiated CAR T to improve avidity. JNJ4528 consists of two BCMA-targeting single-domain fragments with a 4-1BB co-stimulatory domain. Phase Ib CARTITUDE-1 showed early and durable response at low dose of CAR T cells in 90% of patients. Cytokine release syndrome was manageable. ORR was 100% with 76% sCR and 3% PR [203].

CELMoDs are potent novel cereblon E3 Ligase modulators that are specifically designed for rapid and maximal degradation of target proteins called Aiolos and Ikaros. Aiolos and Ikaros are over-expressed in MM cells. The degradation of those proteins leads to specific downregulation of c-Myc and IRF4, resulting in the apoptosis of myeloma cells [204]. Two CELMoDs currently in the clinic are Iberdomide (CC-220) [205] and CC-92480 [206,207]. Both therapeutics have demonstrated enhanced anti-tumor and immune-stimulatory effect in preclinical studies. More importantly, they have shown promising results in overcoming IMiD resistance and a synergistic effect with daratumumab, bortezomib, and DEX [208]. In the phase I study, Iberdomide with DEX demonstrated significant activity with a RR of 31% in patients with six prior therapies [209] and a better AE profile compared to IMiDs. These novel CELMoDs have the potential to overcome clinical resistance and offer a promising treatment for RRMM.

Great progress has been achieved in the past five years in the treatment of MM with the introduction of mAbs. Combination of daratumumab with bortezomib, thalidomide, and DEX has shown great efficacy and progression free durability. Novel combination therapies with lower toxicity profile were approved. Development of novel modalities like ADCs, bsAbs, CELLMoDs, and CAR T cell therapies offer great promise for treatment of MM.

## 5. Emerging Modality in Targeting across Tumor Types

It is now possible to treat many types of cancer based on their specific molecular signature and immune phenotype regardless of the tumor origin. Histology-agnostic therapeutics target the common molecular markers across multiple cancer types, offering a remarkable overall response rate that is durable [210] (Table 13). Keytruda was developed based on specific genetic features of microsatellite instability-high or mismatch-repair deficiency (MSI-H/dMMR) and was approved in 2017 for the treatment of solid tumors. MSI-H is a biomarker for cancer cells with high number of mutations within tracts of microsatellite DNAs and dMMR is the inability of cancer cells to repair mistakes introduced during cell division. Tumors with deficiency in DNA mismatch repair should produce a large amount of neoantigens that could trigger immune responses. Therefore, these cancers are more sensitive to immune checkpoint blockade, regardless of the tumor origins [211,212]. In June 2020, Keytruda was awarded the accelerated approval as a histology-agnostic drug for treatment of Tumor Mutational Burden-High (TMB-H) solid tumors. On this note, Genentech and Eli Lilly are evaluating their PD-L1 antibodies, atezolizumab and LY3300054, in solid tumors with and without MSI-H biomarker [212].

Vitrakvi (larotrectinib) and Rozlytrek (entrectinib) target NTRK fusions. The success of these therapies is due to the fact that NTRK is the single dominant oncogenic driver in fusion positive cancers [213]. Pharmaceutical industry is heavily investing in the histology-agnostic agents as part of oncology pipeline. Tumor-agnostic therapies for cancer patients with biomarkers *RET* fusion, HER2, *FGFR* mutations, *KRAS* mutations, *ROS1*, *ALK*, *G12C,* neuregulin 1 (*NRG1*) fusion [210,212,214], PD-L1 overexpression, and APOBEC alteration [212] are under consideration. Retevmo (selpercatinib) was approved in May 2020 for the treatment of *RET* fusion-positive NSCLC, *RET*-mutant medullary thyroid cancer (MTC), and *RET* fusion-positive thyroid cancer. In the same month, FDA granted breakthrough approval to Enhertu (fam-trastuzumab deruxtecan-nxki), a HER2-directed ADC for NSCLC and gastric cancer patients with HER2 expression in their tumors.

Early detection of different cancer types for immediate interventions might be imminent. A few studies have shown promising results in predicting the occurrence of cancer using ctDNA and their methylation signatures [215]. An algorithm was used to access DNA methylation patterns in the ctDNA collected from 2482 cancer patients and 4207 healthy individuals [216]. The test detected tumor signatures for more than 50 cancer types at different stages (non-metastatic and metastatic). The sensitivity of the assay was 39–69% for patients in early-stage cancer (stage I and II) and 83–92% in patients in advanced stages (stage III and IV). The limitations of this assay include the false-positive rate and low sensitivity due to the lack of ctDNA in the circulation [217,218]. In Taizhou longitudinal study, 605 asymptomatic individuals were tested by PanSeer, a noninvasive blood test based on ctDNA methylation [219]. Within four years, 191 individuals that were asymptomatic at the time of testing were diagnosed with stomach, esophageal, colorectal, lung, or liver cancer. Overall, the study showed a 95% accuracy for detection of cancer in asymptomatic individuals.

## 6. Concluding Remarks

In this review, we have summarized and discussed the current and upcoming therapies for oncology that focus on novel biological targets, new mechanisms of action, and emerging technologies. Target-specific therapies and immunotherapies have shown groundbreaking results in cancer treatment. T cell stimulating therapy include the checkpoint inhibitors or agonist antibodies to improve T cell activity, the utilization of bsAbs to redirect T cells to tumor cells, autologous T cell ex vivo activation combined with bispecific antibodies, CAR T cells with engineered antibodies replacing TCRs, and T cells expressing CD16a with anti-tumor antibodies (e.g., Anti-CD20, or anti-BCMA). Use of Keytruda showed that tumors can be treated based on their genetic profile rather than site-of-origin. Combination drugs have enabled specific targeting of proteins, cells, and tissues. Conjugations or fusions of small molecules, antibodies, peptides, oligonucleotides, lipids, or carbohydrates to mAb have been explored in various stages of preclinical and clinical development for different cancers with many of them entering clinic. In addition, emerging technologies, such as single-cell transcriptomics, machine learning, and stem-cell based human organoids, have enabled acquisition of a vast amount of high-dimensional data in both discovery and development stages that can enhance our understanding of the disease.

## Figures and Tables

**Table 1:** Approved small molecules by the Center for Drug Evaluation and Research (CDER) of the U.S. Food and Drug Administration (FDA) in oncology (2015–2020).

Approval Year	Trade Name	Drug Name	Sponsor	Properties	Indication	Dosage Form	FDA Review
2015	Ibrance	Palbociclib	Pfizer	CDK4 and CDK6 inhibitor	Advanced (metastatic) breast cancer	Capsule	P,B,A
	Lenvima	Lenvatinib	Eisai	VEGFR inhibitor	Progressive, differentiated thyroid cancer (DTC) whose disease progressed despite receiving radioactive iodine therapy (radioactive iodine refractory disease).	Capsule	P,O
	Farydak	Panobinostat	Novartis	Histone deacetylase inhibitor	Multiple myeloma	Capsule	P,O,A
	Odomzo	Sonidegib	Novartis	Smoothened inhibitor	Locally advanced basal cell carcinoma that has recurred following surgery or radiation therapy, or who are not candidates for surgery or radiation therapy.	Capsule	S
	Lonsurf	Trifluridine and tipiracil	Taiho	Thymidine phosphorylase inhibitor plus a nucleoside metabolic inhibitor	An advanced form of colorectal cancer who are no longer responding to other therapies	Tablet	S
	Yondelis	Trabectedin	Johnson & Johnson	Alkylating drug	Specific soft tissue sarcomas (STS) – liposarcoma and leiomyosarcoma – that cannot be removed by surgery (unresectable) or is advanced (metastatic).	Injection	P,O
	Cotellic	Cobimetinib	Genentech	MEK inhibitor	To be used in combination with vemurafenib to treat advanced melanoma that has spread to other parts of the body or can’t be removed by surgery, and that has a certain type of abnormal gene (BRAF V600E or V600K mutation)	Tablet	P,O
	Tagrisso	Osimertinib	AstraZeneca	EGFR inhibitor	Non-small cell lung cancer	Tablet	P,O,B,A
	Ninlaro	Ixazomib	Takeda	Oral proteasome inhibitor	Multiple myeloma who have received at least one prior therapy	Capsule	P,O
	Alecensa	Alectinib	Roche	ALK inhibitor	ALK-positive lung cancer	Capsule	P,O,B,A
2016	Venclexta	Venetoclax	AbbVie	BCL-2 inhibitor	For chronic lymphocytic leukemia in patients with a specific chromosomal abnormality	Tablet	P,O,B,A
	Axumin	Fluciclovine F 18	Blue Earth	Radioactive diagnostic	A new diagnostic imaging agent to detect recurrent prostate cancer	Injection	P
	NETSPOT	Gallium Ga 68 dotatate	Advanced Accelerator Applications	Radioactive diagnostic	A diagnostic imaging agent to detect rare neuroendocrine tumors	Injection	P,O
	Rubraca	Rucaparib	Clovis Oncology	PARP inhibitor	A certain type of ovarian cancer	Tablet	P,O,B,A
2017	Kisqali	Ribociclib	Novartis	CDK4/6 inhibitor	Postmenopausal women with a type of advanced breast cancer	Tablet	P,B
	Zejula	Niraparib	Tesaro	PARP inhibitor	For the maintenance treatment for recurrent epithelial ovarian, fallopian tube or primary peritoneal cancers	Capsule	P,O,B
	Alunbrig	Brigatinib	Ariad Pharmaceuticals/ Takeda	ALK inhibitor	Anaplastic lymphoma kinase (ALK)-positive metastatic non-small cell lung cancer (NSCLC) who have progressed on or are intolerant to crizotinib	Tablet	P,O,B,A
	Rydapt	Midostaurin	Novartis	FLT3 inhibitor	Acute myeloid leukemia, advanced systemic mastocytosis	Capsule	P,O,B
	Nerlynx	Neratinib maleate	Puma Biotechnology	EGFR, HER2 and HER4 irreversible kinase inhibitor	To reduce the risk of breast cancer returning	Tablet	S
	Idhifa	Enasidenib	Celgene/Agios	IDH2 inhibitor	Relapsed or refractory acute myeloid leukemia	Tablet	P,O
	Aliqopa	Copanlisib	Bayer	PI3Kα/δ inhibitor	Relapsed follicular lymphoma	Injection	P,O,A
	Verzenio	Abemaciclib	Eli Lilly	CDK4/6 inhibitor	Advanced or metastatic breast cancers	Tablet	P,B
	Calquence	Acalabrutinib	AstraZeneca/Acerta Pharma	BTK inhibitor	Mantle cell lymphoma	Capsule	P,O,B,A
2018	Erleada	Apalutamide	Johnson & Johnson	Androgen receptor inhibitor	Prostate cancer	Tablet	P
	Braftovi	Encorafenib	Array BioPharma	BRAF inhibitor	Unresectable or metastatic melanoma	Capsule	S,O
	Mektovi	Binimetinib	Array BioPharma	MEK inhibitor	Unresectable or metastatic melanoma	Tablet	S,O
	Tibsovo	Ivosidenib	Agios Pharmaceuticals	IDH1 inhibitor	Relapsed or refractory acute myeloid leukemia	Tablet	P,O
	Copiktra	Duvelisib	Verastem	PI3K inhibitor	Relapsed or refractory chronic lymphocytic leukemia, small lymphocytic lymphoma, and follicular lymphoma	Capsule	P,O,A
	Vizimpro	Dacomitinib	Pfizer	EGFR inhibitor	Metastatic non-small-cell lung cancer	Tablet	P,O
	Talzenna	Talazoparib	Pfizer	PARP inhibitor	Metastatic breast cancer	Capsule	P
	Lorbrena	Lorlatinib	Pfizer	ALK and ROS1 inhibitor	Anaplastic lymphoma kinase (ALK)-positive metastatic non-small cell lung cancer	Tablet	P,O,B,A
	Daurismo	Glasdegib	Pfizer	Hedgehog pathway inhibitor	Acute myeloid leukemia or high-risk myelodysplastic syndrome	Tablet	P,O
	Vitrakvi	Larotrectinib	Loxo Oncology/Bayer	TRKA, TRKB and TRKC inhibitor	Metastatic solid tumors with NTRK-fusion proteins	Capsule	P,O,B,A
	Xospata	Gilteritinib	Catalyst Pharmaceuticals	Potassium channel blocker	Acute myeloid leukemia	Tablet	P,O,B
2019	Balversa	Erdafitinib	Janssen/J&J	FGFR inhibitor	Locally advanced or metastatic bladder cancer	Tablet	P,B,A
	Piqray	Alpelisib	Novartis	PI3K inhibitor	Advanced or metastatic breast cancer	Tablet	P
	Xpovio	Selinexor	Karyopharm Therapeutics	XPO1 inhibitor	Relapsed or refractory multiple myeloma	Tablet	P,O,A
	Nubeqa	Darolutamide	Bayer	Androgen receptor inhibitor	Non-metastatic prostate cancer	Tablet	P
	Turalio	Pexidartinib	Daiichi Sankyo	CSF1R, KIT and FLT3 inhibitor	Symptomatic tenosynovial giant cell tumor	Capsule	P,O,B
	Rozlytrek	Entrectinib	Roche	TRKA, TRKB, TRKC, ROS1 and ALK inhibitor	Metastatic non-small cell lung cancer and locally advanced or metastatic solid tumors with a specific genetic defect	Capsule	P,O,B,A
	Brukinsa	Zanubrutinib	BeiGene	BTK inhibitor	Mantle cell lymphoma	Capsule	P,O,B,A
9/2020	Ayvakit	Avapritinib	Blueprint Medicines	PDGFRα -D816V inhibitor	Unresectable or metastatic gastrointestinal stromal tumor (GIST)	Tablet	P,O
	Tazverik	Tazemetostat	Epizyme Inc.	Methyltransferase inhibitor	Epithelioid sarcoma	Tablet	P,O
	Tukysa	Tucatinib	Seattle Genetics	HER2 tyrosine kinase inhibitor	Advanced unresectable or metastatic HER2-positive breast cancer	Tablet	P,O
	Pemazyre	Pemigatinib	Incyte Corp	FGFR1 kinase inhibitor	Cholangiocarcinoma, a rare form of cancer that forms in bile ducts	Tablet	P,O
	Tabrecta	Capmatinib	Novartis	MET kinase inhibitor	Non-small cell lung cancer	Tablet	P,O
	Retevmo	Selpercatinib	Loxo/Eli Lilly	RET kinase inhibitor	Lung and thyroid cancers	Capsule	P,O
	Qinlock	Ripretinib	Deciphera Pharms	Proto-oncogene receptor tyrosine kinase (KIT) and platelet derived growth factor receptor A (PDGFRA) kinase inhibitor	Advanced gastrointestinal-stromal tumors	Tablet	P,O
	Cerianna	Fluoroestrdiol F18	Zionexa	Radioactive diagnostic agent	Diagnostic imaging agent for certain patients with breast cancer	Solution	S
	Zepzelca	Lurbinectedin	Jazz	Selective oncogenic transcription inhibitor	Metastatic small cell lung cancer	Powder (i.v.)	P,O
	Inqovi	Decitabine and cedazuridine	Astex Pharmaceuticals	A combination of decitabine, a nucleoside metabolic inhibitor, and cedazuridine, a cytidine deaminase inhibitor	Myelodysplastic syndromes	Tablet	P,O
	Gavreto	Pralsetinib	Blueprint Medicines	RET kinase inhibitor	Non-small lung cancer	Capsule	P, O

Source: Drugs@FDA. S: standard; P: priority; B: breakthrough; A: accelerated; O: orphan.

**Table 2:** Novel monoclonal antibody (mAb) approved by CDER of FDA for oncology (2015–2020).

Approval Year	Trade Name	Drug Name	Sponsor	Properties	Indication	FDA Review
2015	Unituxin	Dinutuximab	United Therapeutics	GD2-binding mAb	High-risk neuroblastoma	P,O
	Darzalex	Daratumumab	Johnson and Johnson	CD38-directed mAb	Multiple myeloma who have received at least three prior treatments.	P,O,B,A
	Portrazza	Necitumumab	Eli Lilly	EGFR antagonist	Advanced (metastatic) squamous non-small cell lung cancer (NSCLC) who have not previously received medication specifically for treating their advanced lung cancer	S,O
	Empliciti	Elotuzumab	Bristol-Myers Squibb	SLAMF7-directed mAb	Multiple myeloma who have received one to three prior medications	P,O,B
2016	Tecentriq	Atezolizumab	Genentech	PD-L1 inhibitor	Urothelial carcinoma, the most common type of bladder cancer	P,B,A
	Lartruvo	Olaratumab	Eli Lilly	PDGFRα-blocking antibody	Certain types of soft tissue sarcoma	P,O,B,A
2017	Bavencio	Avelumab	Merck KGaA/Pfizer	PD-L1-blocking antibody	Metastatic Merkel cell carcinoma	P,O,B,A
	Imfinzi	Durvalumab	AstraZeneca	PD-L1-blocking antibody	Locally advanced or metastatic urothelial carcinoma	P,B,A
2018	Poteligeo	Mogamulizumab-kpkc	Kyowa Hakko Kirin	CCR4 antibody	Relapsed or refractory mycosis fungoides or Sézary syndrome	P,O,B
	Libtayo	Cemiplimab-rwlc	Regeneron/Sanofi	PD-1 antibody	Cutaneous squamous cell carcinoma	P,B
9/2020	Sarclisa	Isatuximab	Sanofi Aventis US	CD38-directed cytolytic antibody	Multiple myeloma	S,O
	Monjuvi	Tafasitamab-cxix	Morphosys US Inc	CD19-directed cytolytic antibody	Relapsed or refractory diffuse large B-cell lymphoma	S,O

Source: Drugs@FDA. Dosage form is injection for all mAb drugs. S: Standard; P: Priority; B: Breakthrough; A: Accelerated; O: Orphan.

**Table 3:** All approved antibody-drug conjugates (ADCs) by CDER of FDA are for oncology indications.

Approval Year	Trade Name	Drug Name	Sponsor	Properties	Indication
2001 *	Mylotarg	Gemtuzumab ozogamicin	Wyeth Pharmas	CD33-directed ADC	CD33-positive acute myeloid leukemia
2002	Zevalin	Ibritumomab tiuxetan	Spectrum Pharms	CD20-directed ADC	Relapsed or refractory, low-grade or follicular B-cell non-Hodgkin’s lymphoma
2011	Adcetris	Brentuximab vedotin	Seattle Genetics	CD30-directed ADC	Hodgkin lymphoma and systemic anaplastic large cell lymphoma
2013	Kadcyla	Trastuzumab emtansine	Genetech	HER2-directed ADC	HER2-positive, metastatic breast cancer
2017	Besponsa	Inotuzumab ozogamicin	Pfizer	CD22-directed ADC	To treat adults with relapsed or refractory acute lymphoblastic leukemia
2018	Lumoxiti	Moxetumomab pasudotox-tdfx	AstraZeneca	CD22-directed ADC	Hairy cell leukemia
2019	Polivy	Polatuzumab vedotin	Roche	CD79b- directed ADC	Relapsed or refractory diffuse large B-cell lymphoma
	Padcev	Enfortumab vedotin-ejfv	Astellas	Nectin-4-directed ADC	Refractory bladder cancer
	Enhertu	Fam-trastuzumab deruxtecan-nxki	Daiichi Sankyo/AstraZeneca	HER2-directed ADC	Metastatic breast cancer
9/2020	Trodelvy	Sacituzumab govitecan-hziy	Immunomedics	TROP2-directed ADC	To treat adult patients with metastatic triple-negative breast cancer who received at least two prior therapies for metastatic disease
	Blenrep	Belantamab mafodotin-blmf	GlaxoSmithKline	B-cell maturation antigen (BCMA)-directed ADC	To treat multiple myeloma

Source: Drugs@FDA. * Mylotarg was withdrawn in 2010, and re-approved in 2017. Dosage form is injection for all ADC drugs.

**Table 4:** Select approved cell and gene therapy for oncology by CBER (FDA).

Approval Year	Trade Name	Drug Name	Sponsor	Properties	Indication for Use
2010	PROVENGE	Sipuleucel-T	Dendreon Corporation	Cell-based immunotherapy	Prostate cancer
2015	Imlygic	Talimogene laherparepvec	Amgen	Genetically modified oncolytic virus	Melanoma
2017	Kymriah	Tisagenlecleucel	Novartis	CD19-directed CAR T therapy	B-cell precursor acute lymphoblasticleukemia (ALL)
2017	Yescarta	Axicabtagene ciloleucel	Kite Pharma/Gilead Sciences	CD19-directed CAR T therapy	large B-cell lymphoma
2020	Tecartus	Brexucabtagene autoleucel	Kite Pharma/Gilead Sciences	CD19-directed CAR T therapy	Relapsed/refractory mantle cell lymphoma (r/r MCL)

CBER: the Center for Biologics Evaluation and Research; CAR T: Chimeric antigen receptor T cell; Novel oligonucleotide-based drugs are categorized as novel entities reviewed by CDER.

**Table 5:** Subtypes of breast cancer based on receptor expression and the five-year survival rate.

Breast Cancer Subtype	Prevalence	Receptor	Ki67 Level *	5-Year Survival at Different Stages of Breast Cancer			Treatment Option
				Localized	Regional	Distant	
Luminal A	50–60%	HR+ (ER+ and/or PR+), Her2−	Low	100%	89.9	30.4%	Hormonal therapy, CDK inhibitor, often resistant to chemotherapy
Luminal B	15–20%	HR+ (ER+ and/or PR+), HER2−/+	High	98.7%	89.5%	43.5%	Chemotherapy, CDK inhibitor, HER2 targeted, and hormonal therapy
HER2/neu+	15–20% Non-luminal	HER2+	High	96.1%	81.7%	36.8%%	Chemotherapy and HER2 targeted therapy
Basal-like (80% TNBC)	8–37% ductal	ER-, PR-, HER2-	High	91.1%	65%	11.5%	Chemotherapy, immunotherapy, antibody-drug conjugate

* Ki67 (Prognostic marker for Breast cancer): level indicates cell proliferation rate. CDK: cyclic-dependent kinase; +: positive; −: negative; -/+: either positive or negative.

**Table 6:** Approved ADCs for HER2-positive breast cancer.

Drug Name	Antibody	Cytotoxic Payload	Linkage	DAR	Target Population	Efficacy Data
Kadcyla or T-DM1	Trastuzumab	Maytansinoid, DM1 (microtubule inhibitor)	Stable thioether linkage	3.5	HER2+ advanced breast cancer	In T-DM1,OS 30.9 months; PFS 9 months compared to 25.1 months and 6.4 months in control arm (lapatinib +capecitabine)
					As adjuvant therapy in HER2+ early breast cancer	Three-year disease free survival in T-DM1 was 88.3% vs. 77% in trastuzumab
Enhertu or T-Dxd	Trastuzumab	Topoisomerase I inhibitor (DXd)	Tetrapeptide based cleavable linker	7.7	Metastatic or unresectable HER2+	In T-Dxd arm,PFS-16.4 months; ORR-60.3% (4.3% CR and 56% PR) CNS subgroup: ORR 58.3% and PFS was 18.1 months

HER2+: HER2-positive; Dxd: Dexatecan derivative.

**Table 8:** Novel targets in clinical trial against TNBC.

Target	Drug Name	Trial/Combination	Efficacy Data	Reference
*PIK3CA*, *AKT1*, *PTEN* aberration	Ipatasertib (AKT inhibitor)	LOTUS, phase II/ipatasertib + paclitaxel vs. placebo+ paclitaxel	For normal PTEN, OS-28 months vs. 17.1 months; one-year survival was 85% vs. 68%; For low PTEN, OS 23.1 months vs. 15.8 months; one-year survival 79% vs. 64%.	[78,79]
		Phase Ib/ipatasertib + atezolizumab and paclitaxel as first-line therapy	Triplet regimen showed ORR 73% and anti-tumor activity irrespective of tumor biomarker status	[80]
	Capivasertib (AKT inhibitor)	PAKT trial, phase II/capivasertib+ paclitaxel vs. placebo+ paclitaxel	In ITT population, PFS and OS 5.9 months and 19.1 months vs. 4.2 months and 12.6 months; in *PIK3CA*, *AKT1* or *PTEN* alteration grp: ORR and PFS was 35.3% and 9.3 months vs. 18.2% and 3.7 months; AE grade 3 or 4 was 54.4% vs. 25.7%	[81]
Androgen Receptor (AR) (12–55% of TNBC) [82]	Enzalutamide	Phase II	PFS, OS was 2.9 months, 12 months in ITT population and 3.3 months and 17.6 months in patients with ≥ 10% AR+ tumors	[83]
Histone deacetylase (HDAC)	Panobinostat (HDAC inhibitor)	Phase I/panobinostat + letrozole vs. letrozole +placebo		[84]

OS: Overall survival; ITT: Intend to treat; PFS: Progression free survival.

**Table 11:** Patient subtypes in multiple myeloma.

Abnormality Type	Subtype	Subtype Classification	Gene/Chromosome Affected	Percentage of MM Patients
Primary abnormalities	IgH translocation	t(11;14)	CCDN1, cyclic D1, CD20	15–20%
		t(6:14)	*CCND3*	2%
		t(4;14)	Deregulation of *FGFR3* and *MMSET*	10–15%
		t(14;16)	Overexpression of the *c-MAF* proto-oncogene and lack of CD56 expression	2–5%
		t(14;20)	Upregulation of the *MAFB* oncogene	1%
	Trisomies	Hyperploidy	Gain of the odd-numbered chromosomes 3, 5, 7, 9, 11, 15, 19, and 21, IgG Kappa	50%
		Non- Hyperploidy		45%
Secondary abnormalities	Translocation	Myc	Partner loci include *IGH*, *IGL*, *IGK*, *FAM46C*, *FOXO3*, and *BMP6*	15–20%
	Copy number alteration	1q21 gain	*CKS1B*	35–40%
		1p deletion	Common deletion: 1p32 (*CDKN2C*), 1p22, and 1p12	30%
		13q	Deletion of the long arm of chromosome 13, interstitial deletions 13q14.11-13q14.3	45–50%
		17p	*TP53* deletion	10%

**Table 12:** Overview of daratumumab approvals for multiple myeloma.

Drug Name (Year) Approved	Trial Name	Efficacy Data	Target Patient	Reference
Daratumumab monotherapy (2015)	GEN501	ORR 36%; PFS 5.6 months; one-year survival in 77% responder; one-year disease-free survival in 65%	RRMM pretreated with at least 3 lines of therapy (PI, IMiD or double refractory to an IMiD and PI	[173]
	SIRIUS	ORR 30.4%; OS 20.5 months; three-year OS rate 36.5%		
Daratumumab with bortezomib and DEX (2016)	CASTOR	PFS NR vs. 7.1 (Vd); Minimum MRD; Risk of progression reduced by 61%	MM patients who have received at least 1 prior therapy	[174]
Daratumumab with lenalidomide and DEX (2016)	POLLUX	Risk of progression reduced by 63%; MRD 30.4% vs. 5.3%; PFS–NR vs. 18.4 months; Prolonged median time to next therapy from 23.1 months to 50.6 months	RRMM	[174]
Daratumumab with pomalidomide, and DEX (2017)	EQUULEUS	ORR 60% (58% in double-refractory patients); Among responders at follow-up of 13.1 months PFS 8.8 months and OS 17.5 months; 12-month survival rate 66%; Higher MRD negativity rate 29% at a threshold of 10^−5^	MM patient who received at least 2 prior therapy including PI and lenalidomide	[175]
Daratumumab with melphalan-bortezomib-prednisone (D-MVP) (2018)	ALCYONE	ORR 90% (PR-19.7%, VGPR- 28.6%, CR 24.6%, SCR-18%) vs. 73.9% (24.2%, 25.2%, 17.4%, 7%); Response lasted for 18 months 71.6% vs. 50.2%; MRD negativity rate 22.3% vs. 6.2%; 50% reduction in disease progression; Median PFS NR vs. 18.1 months	SoC for NDMM as combination therapy for patient not eligible for ASCT	[164]
Daratumumab with lenalidomide plus DEX (D-Rd) (2019)	MAIA	Median follow up of 28 months showed PFS NR vs. 31.9 months; VGPR 79% vs. 53%; CR 47.6% vs. 24.9%; MRD negativity (24.2 vs. 7.3%); Risk of progression reduced by 44%		[164]
Daratumumab with bortezomib, thalidomide, and DEX (DVTd) during induction (4 cycles) and consolidation (2 cycles) (2019)	CASSIOPEIA	sCR rate 28.9% vs. 20.3% in VTd group MRD negativity 64% vs. 44% CR- 36% vs. 26% Reduction in risk of death 53% compared to VTd	NDMM patient who is eligible for ASCT This treatment regiment given before and after ASCT	[176]
Daratumumab with carfilzomib plus DEX	CANDOR EQUULEUS	Median PFS was not reached for the DKd arm and was 15.8 months for the Kd arm ORR was 81%, with duration response of 27.5 months	RRMM who have received 1 to 3 lines of therapy	[177]
Daratumumab with hyaluronidase-fihj (2020)	COLUMBA (SC) D vs. (IV) D	ORR in 108 (41%) patients in the subreast cancerutaneous group and 96 (37%) in the intravenous group; ARR 13% in SC group and 34% in IV group	NDMM and transplant-ineligible patients and RRMM	[172]
	PLEIADES	ORR 88.1% in the D-VMP cohort, 90.8% for the D-Rd arm and 97% for the D-VRd arm		

PR: Partial response; VGPR: Very good partial response; CR: Complete response; sCR: Stringent complete response; ARR: Administration related reaction; ORR: Overall response rate; PFS: Progression free survival.

**Table 13:** Approved histology-agnostic therapeutics.

Molecular Alteration	Trade Name (Drug Name)	Drug Properties	Targeted Tumor Types in Trials and Approval Year
Microsatellite instability-high/mismatch-repair deficiency (MSI-H/dMMR)	Keytruda (pembrolizumab)	PD-1 inhibitor antibody	15 tumor types (2017)
Tumor Mutational Burden-High (TMB-H)	Keytruda (pembrolizumab)	PD-1 inhibitor antibody	10 tumor types (2020)
NTRK fusions	Vitrakvi (larotrectinib)	Pan-tropomyosin-related kinase (TRK) inhibitor	17 tumor types (2018)
	Rozlytrek (entrectinib)	Tyrosine-kinase inhibitor (TKI) targeting ROS1, ALK, TRKA, TRKB and TRKC	10 tumor types (2019)

Source: drug@FDA.

## Supplementary Materials

The following are available online at https://www.mdpi.com/1422-0067/22/4/2008/s1, Figure S1: Comparison of global therapeutic sales of the top selling drugs (>1 billion USD) in oncology in 2015 vs. 2019; Table S1: Novel drug approvals including cell/gene therapy summarized by therapeutic areas (2015–2020).

## Abbreviations

FDA	U.S. Food and Drug Administration
CDER	immunohistochemistry
CBER	Center for Biologics Evaluation and Research
WHO	World Health Organization
mAb	Monoclonal antibody
bsAb	Bispecific antibody
ADC	Antibody-drug conjugate
CAR T	Chimeric antigen receptor T cell
AAV	adeno-associated virus
ON	Oligonucleotide
ASO	Antisense oligonucleotide
siRNA	Short interfering RNA
CRISPR	clustered regularly interspaced short palindromic repeats
PFS	Progression-free-survival
HER2	Human epidermal growth factor receptor 2
TNBC	Triple-negative breast cancer
BRCA	Breast cancer gene
NSCLC	Non-small lung cancer
SCLC	Small cell lung cancer
ASCT	Autologous stem cell transformation
PI	Proteasome inhibitor
IMiDs	Immunomodulatory drugs
DEX	Dexamethasone
NDMM	Newly-diagnosed multiple myeloma
RRMM	Relapsed/refractory myeloma
MRD	Minimal residual disease

## References

[1] American Cancer Society (ACS) (2018). Global Cancer Facts & Figures.

[2] IQVIA Global Oncology Trends 2019. Therapeutics, Clinical Development and Health System Implications. www.iqvia.com.

[3] Lipinski C.A., Lombardo F., Dominy B.W., Feeney P.J. (2001). Experimental and computational approaches to estimate solubility and permeability in drug discovery and development settings. Adv. Drug Deliv. Rev..

[4] Yang W., Gadgil P., Krishnamurthy V.R., Landis M., Mallick P., Patel D., Patel P.J., Reid D.L., Sanchez-Felix M. (2020). The Evolving Druggability and Developability Space: Chemically Modified New Modalities and Emerging Small Molecules. AAPS J..

[5] Ottis P., Toure M., Cromm P.M., Ko E., Gustafson J.L., Crews C.M. (2017). Assessing Different E3 Ligases for Small Molecule Induced Protein Ubiquitination and Degradation. ACS Chem. Biol..

[6] Neklesa T., Snyder L.B., Willard R.R., Vitale N., Pizzano J., Gordon D.A., Bookbinder M., Macaluso J., Dong H., Ferraro C. (2019). ARV-110: An oral androgen receptor PROTAC degrader for prostate cancer. J. Clin. Oncol..

[7] Flanagan J.J., Qian Y., Gough S.M., Andreoli M., Bookbinder M., Cadelina G., Bradley J., Rousseau E., Willard R., Pizzano J. (2019). Abstract P5-04-18: ARV-471, an oral estrogen receptor PROTAC degrader for breast cancer. Cancer Res..

[8] Leung D., Wurst J.M., Liu T., Martinez R.M., Datta-Mannan A., Feng Y. (2020). Antibody Conjugates-Recent Advances and Future Innovations. Antibodies.

[9] Labrijn A.F., Janmaat M.L., Reichert J.M., Parren P. (2019). Bispecific antibodies: A mechanistic review of the pipeline. Nat. Rev. Drug Discov..

[10] Jovcevska I., Muyldermans S. (2020). The Therapeutic Potential of Nanobodies. BioDrugs.

[11] Baliga R., Li K., Manlusoc M., Hinton P.R., Ng D.C., Tran M.H., Shan B., Lu H., Saini A., Rahman S. (2020). A Bispecific IgM Antibody Format for Enhanced T cell-Dependent Killing with Minimal Cytokine Release. Cancer Res..

[12] Lau J.L., Dunn M.K. (2018). Therapeutic peptides: Historical perspectives, current development trends, and future directions. Bioorg. Med. Chem..

[13] Yu J.X., Upadhaya S., Tatake R., Barkalow F., Hubbard-Lucey V.M. (2020). Cancer cell therapies: The clinical trial landscape. Nat. Rev. Drug Discov..

[14] Businesswire U.S. FDA Approves Kite’s Tecartus™, the First and Only CAR T Treatment for Relapsed or Refractory Mantle Cell Lymphoma. Businesswire. www.businesswire.com.

[15] Fukuhara H., Ino Y., Todo T. (2016). Oncolytic virus therapy: A new era of cancer treatment at dawn. Cancer Sci..

[16] Matveeva O.V., Kochneva G.V., Zainutdinov S.S., Ilyinskaya G.V., Chumakov P.M. (2018). Oncolytic Paramyxoviruses: Mechanism of Action, Preclinical and Clinical Studies. Mol. Biol. (Mosk.).

[17] Kaufman H.L., Kohlhapp F.J., Zloza A. (2016). Oncolytic viruses: A new class of immunotherapy drugs. Nat. Rev. Drug Discov..

[18] Russell L., Peng K.W. (2018). The emerging role of oncolytic virus therapy against cancer. Chin. Clin. Oncol..

[19] Valeur E., Gueret S.M., Adihou H., Gopalakrishnan R., Lemurell M., Waldmann H., Grossmann T.N., Plowright A.T. (2017). New Modalities for Challenging Targets in Drug Discovery. Angew. Chem. Int. Ed. Engl..

[20] Nair J.K., Willoughby J.L., Chan A., Charisse K., Alam M.R., Wang Q., Hoekstra M., Kandasamy P., Kel’in A.V., Milstein S. (2014). Multivalent N-acetylgalactosamine-conjugated siRNA localizes in hepatocytes and elicits robust RNAi-mediated gene silencing. J. Am. Chem. Soc..

[21] Yu R.Z., Graham M.J., Post N., Riney S., Zanardi T., Hall S., Burkey J., Shemesh C.S., Prakash T.P., Seth P.P. (2016). Disposition and Pharmacology of a GalNAc3-conjugated ASO Targeting Human Lipoprotein (a) in Mice. Mol. Nucleic Acids.

[22] Ma Y., Kowolik C.M., Swiderski P.M., Kortylewski M., Yu H., Horne D.A., Jove R., Caballero O.L., Simpson A.J., Lee F.T. (2011). Humanized Lewis-Y specific antibody based delivery of STAT3 siRNA. ACS Chem. Biol..

[23] Ashmore-Harris C., Fruhwirth G.O. (2020). The clinical potential of gene editing as a tool to engineer cell-based therapeutics. Clin. Transl. Med..

[24] Kim H., Kim J.S. (2014). A guide to genome engineering with programmable nucleases. Nat. Rev. Genet..

[25] Katrekar D., Chen G., Meluzzi D., Ganesh A., Worlikar A., Shih Y.R., Varghese S., Mali P. (2019). In vivo RNA editing of point mutations via RNA-guided adenosine deaminases. Nat. Methods.

[26] Nishikura K. (2016). A-to-I editing of coding and non-coding RNAs by ADARs. Nat. Rev. Mol. Cell. Biol..

[27] Reardon S. (2020). Step aside CRISPR, RNA editing is taking off. Nature.

[28] Doudna J.A., Charpentier E. (2014). Genome editing. The new frontier of genome engineering with CRISPR-Cas9. Science.

[29] Wu X., Ma W., Mei C., Chen X., Yao Y., Liu Y., Qin X., Yuan Y. (2020). Description of CRISPR/Cas9 development and its prospect in hepatocellular carcinoma treatment. J. Exp. Clin. Cancer Res..

[30] Stein R. First U.S. Patients Treated with CRISPR as Human Gene-Editing Trials Get Underway. www.npr.org.

[31] Henderson H. CRISPR Clinical Trials: A 2019 Update. Innovative Genomics Institute. innovativegenomics.org.

[32] Rosenbaum L. New Data from First Human Crispr Trials Shows Promising Results. www.forbes.com.

[33] NIH CRISPR Based Clinical trials on Clinicaltrials.gov. https://clinicaltrials.gov/ct2/results?term=CRISPR&recrs=b&recrs=a&recrs=f&recrs=d&age_v=&gndr=&type=&rslt=&Search=Apply.

[34] NIH (2018). Cancer Stat Facts: Female Breast Cancer.

[35] Schmidt M. (2016). Dose-Dense Chemotherapy in Metastatic Breast Cancer: Shortening the Time Interval for a Better Therapeutic Index. Breast Care.

[36] NIH (2016). Cancer Stat Facts: Female Breast Cancer Subtypes.

[37] Mamounas E.P., Russell C.A., Lau A., Turner M.P., Albain K.S. (2018). Clinical relevance of the 21-gene Recurrence Score(^®^) assay in treatment decisions for patients with node-positive breast cancer in the genomic era. NPJ Breast Cancer.

[38] Leone J.P., Leone B.A. (2015). Breast cancer brain metastases: The last frontier. Exp. Hematol. Oncol..

[39] Davies C., Pan H., Godwin J., Gray R., Arriagada R., Raina V., Abraham M., Medeiros Alencar V.H., Badran A., Bonfill X. (2013). Long-term effects of continuing adjuvant tamoxifen to 10 years versus stopping at 5 years after diagnosis of oestrogen receptor-positive breast cancer: ATLAS, a randomised trial. Lancet.

[40] Burstein H.J., Lacchetti C., Anderson H., Buchholz T.A., Davidson N.E., Gelmon K.A., Giordano S.H., Hudis C.A., Solky A.J., Stearns V. (2019). Adjuvant Endocrine Therapy for Women With Hormone Receptor-Positive Breast Cancer: ASCO Clinical Practice Guideline Focused Update. J. Clin. Oncol..

[41] Mauri D., Pavlidis N., Polyzos N.P., Ioannidis J.P. (2006). Survival with aromatase inhibitors and inactivators versus standard hormonal therapy in advanced breast cancer: Meta-analysis. J. Natl. Cancer Inst..

[42] ACS (2018). Breast Cancer Fact and Figure 2017-2018.

[43] D’Souza A., Spicer D., Lu J. (2018). Overcoming endocrine resistance in metastatic hormone receptor-positive breast cancer. J. Hematol. Oncol..

[44] Royce M.E., Osman D. (2015). Everolimus in the Treatment of Metastatic Breast Cancer. Breast Cancer.

[45] Niu Y., Xu J., Sun T. (2019). Cyclin-Dependent Kinases 4/6 Inhibitors in Breast Cancer: Current Status, Resistance, and Combination Strategies. J. Cancer.

[46] FDA (2019). FDA Approves First PI3K Inhibitor for Breast Cancer.

[47] Jones R., Carucci M., Casbard A., Butler R., Alchami F., Bale C., Bezecny P., Joffe J., Moon S., Twelves C. (2019). Capivasertib (AZD5363) plus fulvestrant versus placebo plus fulvestrant after relapse or progression on an aromatase inhibitor in metastatic ER-positive breast cancer (FAKTION): A randomized, double-blind, placebo-controlled, phase II trial. J. Clin. Oncol..

[48] Jiang Z., Li W., Hu X., Zhang Q., Sun T., Cui S., Wang S., Ouyang Q., Yin Y., Geng C. (2019). Tucidinostat plus exemestane for postmenopausal patients with advanced, hormone receptor-positive breast cancer (ACE): A randomised, double-blind, placebo-controlled, phase 3 trial. Lancet Oncol..

[49] ACS Targeted Therapy for Breast Cancer.

[50] FDA (2017). FDA Approves Neratinib for Extended Adjuvant Treatment of Early Stage HER2-Positive Breast Cancer.

[51] FDA (2020). FDA Approves Neratinib for Metastatic HER2-Positive Breast Cancer.

[52] Duchnowska R., Loibl S., Jassem J. (2018). Tyrosine kinase inhibitors for brain metastases in HER2-positive breast cancer. Cancer Treat Rev..

[53] Ramakrishna N., Temin S., Chandarlapaty S., Crews J.R., Davidson N.E., Esteva F.J., Giordano S.H., Gonzalez-Angulo A.M., Kirshner J.J., Krop I. (2014). Recommendations on disease management for patients with advanced human epidermal growth factor receptor 2-positive breast cancer and brain metastases: American Society of Clinical Oncology clinical practice guideline. J. Clin. Oncol..

[54] Heffron T.P. (2016). Small Molecule Kinase Inhibitors for the Treatment of Brain Cancer. J. Med. Chem..

[55] FDA (2020). FDA Approves Tucatinib for Patients with HER2-Positive Metastatic Breast Cancer.

[56] Awada A.H., Brufsky A.M., Saura C., Freedman R.A., Lin N.U., Bebchuk J., Xu F., Hurvitz S. (2020). Abstract P2-20-01: Impact of neratinib on development and progression of central nervous system (CNS) metastases in patients with HER2-positive metastatic breast cancer (MBC): Findings from the NALA, NEfERT-T, and TBCRC 022 trials. J. Cancer Res..

[57] Freedman R.A., Gelman R.S., Anders C.K., Melisko M.E., Parsons H.A., Cropp A.M., Silvestri K., Cotter C.M., Componeschi K.P., Marte J.M. (2019). TBCRC 022: A Phase II Trial of Neratinib and Capecitabine for Patients With Human Epidermal Growth Factor Receptor 2–Positive Breast Cancer and Brain Metastases. J. Clin. Oncol..

[58] Peddi P.F., Hurvitz S.A. (2013). Trastuzumab emtansine: The first targeted chemotherapy for treatment of breast cancer. Future Oncol..

[59] FDA (2019). FDA Approves Ado-Trastuzumab Emtansine for Early Breast Cancer.

[60] Hunter F.W., Barker H.R., Lipert B., Rothé F., Gebhart G., Piccart-Gebhart M.J., Sotiriou C., Jamieson S.M.F. (2020). Mechanisms of resistance to trastuzumab emtansine (T-DM1) in HER2-positive breast cancer. Br. J. Cancer.

[61] Jerusalem G., Park Y.H., Yamashita T., Hurvitz S.A., Chen S., Cathcart J., Lee C., Perrin C. (2020). 138O CNS metastases in HER2-positive metastatic breast cancer treated with trastuzumab deruxtecan: DESTINY-Breast01 subgroup analyses. Ann. Oncol..

[62] NIH (2020). DS-8201a in Pre-Treated HER2 Breast Cancer That Cannot Be Surgically Removed or Has Spread [DESTINY-Breast02].

[63] NIH (2020). DS-8201a versus T-DM1 for Human Epidermal Growth Factor Receptor 2 (HER2)-Positive, Unresectable and/or Metastatic Breast Cancer Previously Treated with Trastuzumab and Taxane [DESTINY-Breast03].

[64] NIH (2020). Trastuzumab Deruxtecan (DS-8201a) versus Investigator’s Choice for HER2-low Breast Cancer That Has Spread or Cannot Be Surgically Removed [DESTINY-Breast04].

[65] Rinnerthaler G., Gampenrieder S.P., Greil R. (2019). HER2 Directed Antibody-Drug-Conjugates beyond T-DM1 in Breast Cancer. Int. J. Mol. Sci..

[66] Sassen A., Rochon J., Wild P., Hartmann A., Hofstaedter F., Schwarz S., Brockhoff G. (2008). Cytogenetic analysis of HER1/EGFR, HER2, HER3 and HER4 in 278 breast cancer patients. Breast Cancer Res..

[67] Skidmore L., Sakamuri S., Knudsen N.A., Hewet A.G., Milutinovic S., Barkho W., Biroc S.L., Kirtley J., Marsden R., Storey K. (2020). ARX788, a Site-specific Anti-HER2 Antibody—Drug Conjugate, Demonstrates Potent and Selective Activity in HER2-low and T-DM1–resistant Breast and Gastric Cancers. J. Mol. Cancer Ther..

[68] Saura C., Thistlethwaite F., Banerji U., Lord S., Moreno V., MacPherson I., Boni V., Rolfo C.D., Vries E.G.E.d., Herpen C.M.L.-V. (2018). A phase I expansion cohorts study of SYD985 in heavily pretreated patients with HER2-positive or HER2-low metastatic breast cancer. J. Clin. Oncol..

[69] Hu X., Zhang J., Ji D., Xia G., Ji Y., Xiong G., Liang X. (2020). Abstract P1-18-16: A phase 1 study of ARX788, a HER2-targeting antibody-drug conjugate, in patients with metastatic HER2-positive breast cancer. J. Cancer Res..

[70] Masuda N., Yonemori K., Takahashi S., Kogawa T., Nakayama T., Iwase H., Takahashi M., Toyama T., Saeki T., Saji S. (2019). Abstract PD1-03: Single agent activity of U3-1402, a HER3-targeting antibody-drug conjugate, in HER3-overexpressing metastatic breast cancer: Updated results of a phase 1/2 trial. J. Cancer Res..

[71] Rugo H.S., Im S.-A., Cardoso F., Cortes J., Curigliano G., Pegram M.D., Musolino A., Bachelot T., Wright G.S., De Laurentiis M. (2020). Abstract GS1-02: Phase 3 SOPHIA study of margetuximab + chemotherapy vs trastuzumab + chemotherapy in patients with HER2+ metastatic breast cancer after prior anti-HER2 therapies: Second interim overall survival analysis. J. Cancer Res..

[72] Isakoff S.J. (2010). Triple-negative breast cancer: Role of specific chemotherapy agents. Cancer J..

[73] Guestini F., McNamara K.M., Ishida T., Sasano H. (2016). Triple negative breast cancer chemosensitivity and chemoresistance: Current advances in biomarkers indentification. Expert Opin. Targets.

[74] Lehmann B.D., Bauer J.A., Chen X., Sanders M.E., Chakravarthy A.B., Shyr Y., Pietenpol J.A. (2011). Identification of human triple-negative breast cancer subtypes and preclinical models for selection of targeted therapies. J. Clin. Investig..

[75] Hubalek M., Czech T., Müller H. (2017). Biological Subtypes of Triple-Negative Breast Cancer. Breast Care.

[76] Shah S.P., Roth A., Goya R., Oloumi A., Ha G., Zhao Y., Turashvili G., Ding J., Tse K., Haffari G. (2012). The clonal and mutational evolution spectrum of primary triple-negative breast cancers. Nature.

[77] LoRusso P.M. (2016). Inhibition of the PI3K/AKT/mTOR Pathway in Solid Tumors. J. Clin. Oncol..

[78] NIH A Study of Ipatasertib in Combination with Paclitaxel as a Treatment for Participants with PIK3CA/AKT1/PTEN-Altered, Locally Advanced or Metastatic, Triple-Negative Breast Cancer or Hormone Receptor-Positive, HER2-Negative Breast Cancer (IPATunity130); NIH: 2020. https://clinicaltrials.gov/ct2/show/NCT03337724.

[79] Peng W., Chen J.Q., Liu C., Malu S., Creasy C., Tetzlaff M.T., Xu C., McKenzie J.A., Zhang C., Liang X. (2016). Loss of PTEN Promotes Resistance to T Cell-Mediated Immunotherapy. Cancer Discov..

[80] Schmid P., Loirat D., Savas P., Espinosa E., Boni V., Italiano A., White S., Singel S.M., Withana N., Mani A. (2019). Abstract CT049: Phase Ib study evaluating a triplet combination of ipatasertib (IPAT), atezolizumab (atezo), and paclitaxel (PAC) or nab-PAC as first-line (1L) therapy for locally advanced/metastatic triple-negative breast cancer (TNBC). Cancer Res..

[81] Killock D. (2020). AKT inhibition improves OS in TNBC. Nat. Rev. Clin. Oncol..

[82] Barton V.N., D’Amato N.C., Gordon M.A., Christenson J.L., Elias A., Richer J.K. (2015). Androgen Receptor Biology in Triple Negative Breast Cancer: A Case for Classification as AR+ or Quadruple Negative Disease. Horm. Cancer.

[83] Traina T.A., Miller K., Yardley D.A., Eakle J., Schwartzberg L.S., O’Shaughnessy J., Gradishar W., Schmid P., Winer E., Kelly C. (2018). Enzalutamide for the Treatment of Androgen Receptor—Expressing Triple-Negative Breast Cancer. J. Clin. Oncol..

[84] Tan W.W., Allred J.B., Moreno-Aspitia A., Northfelt D.W., Ingle J.N., Goetz M.P., Perez E.A. (2016). Phase I Study of Panobinostat (LBH589) and Letrozole in Postmenopausal Metastatic Breast Cancer Patients. Clin. Breast Cancer.

[85] Mina A., Yoder R., Sharma P. (2017). Targeting the androgen receptor in triple-negative breast cancer: Current perspectives. Oncol. Targets.

[86] Sharifi M., Wisinski K., Burkard M., Tevaarwerk A., Tamkus D., Chan N., Truica C., Danciu O., Hoskins K., O’Regan R. (2019). Abstract OT1-02-01: Phase I trial of bicalutamide and ribociclib in androgen receptor-positive triple negative breast cancer. J. Cancer Res..

[87] Reese J., Babbs B., Christenson J., Spoelstra N., Elias A., Eisner J., Baskin-Bey E., Gertz J., Richer J. (2019). Abstract P5-05-05: Targeting the androgen receptor with seviteronel, a CYP17 lyase and AR inhibitor, in triple negative breast cancer. J. Cancer Res..

[88] Sikandar B., Qureshi M.A., Naseem S., Khan S., Mirza T. (2017). Increased Tumour Infiltration of CD4+ and CD8+ T-Lymphocytes in Patients with Triple Negative Breast Cancer Suggests Susceptibility to Immune Therapy. Asian Pac. J. Cancer Prev..

[89] Li Z., Qiu Y., Lu W., Jiang Y., Wang J. (2018). Immunotherapeutic interventions of Triple Negative Breast Cancer. J. Transl. Med..

[90] FDA *FDA Approves Atezolizumab for PD-L1 Positive Unresectable Locally Advanced or Metastatic Triple-Negative Breast Cancer*; 2019. https://www.fda.gov/drugs/drug-approvals-and-databases/fda-approves-atezolizumab-pd-l1-positive-unresectable-locally-advanced-or-metastatic-triple-negative.

[91] Cortes J., Cescon D.W., Rugo H.S., Nowecki Z., Im S.-A., Yusof M.M., Gallardo C., Lipatov O., Barrios C.H., Holgado E. (2020). KEYNOTE-355: Randomized, double-blind, phase III study of pembrolizumab + chemotherapy versus placebo + chemotherapy for previously untreated locally recurrent inoperable or metastatic triple-negative breast cancer. J. Clin. Oncol..

[92] Long L., Zhang X., Chen F., Pan Q., Phiphatwatchara P., Zeng Y., Chen H. (2018). The promising immune checkpoint LAG-3: From tumor microenvironment to cancer immunotherapy. Genes Cancer.

[93] FDA (2020). FDA Approves New Therapy for Triple Negative Breast Cancer That Has Spread, Not Responded to Other Treatments.

[94] Nagayama A., Vidula N., Ellisen L., Bardia A. (2020). Novel antibody-drug conjugates for triple negative breast cancer. Adv. Med. Oncol..

[95] Han H., Diab S., Alemany C., Basho R., Brown-Glaberman U., Meisel J., Pluard T., Cortes J., Dillon P., Ettl J. (2020). Abstract PD1-06: Open label phase 1b/2 study of ladiratuzumab vedotin in combination with pembrolizumab for first-line treatment of patients with unresectable locally-advanced or metastatic triple-negative breast cancer. J. Cancer Res..

[96] Cunningham D.L., Sarhan A.R., Creese A.J., Larkins K.P.B., Zhao H., Ferguson H.R., Brookes K., Marusiak A.A., Cooper H.J., Heath J.K. (2020). Differential responses to kinase inhibition in FGFR2-addicted triple negative breast cancer cells: A quantitative phosphoproteomics study. Sci. Rep..

[97] NIH (2020). Cisplatin Plus Romidepsin & Nivolumab in Locally Recurrent or Metastatic Triple Negative Breast Cancer (TNBC).

[98] NIH Entinostat, Nivolumab, and Ipilimumab in Treating Patients With Solid Tumors That Are Metastatic or Cannot Be Removed by Surgery or Locally Advanced or Metastatic HER2-Negative Breast Cancer; NIH: 2020. https://clinicaltrials.gov/ct2/show/NCT02453620.

[99] Hwang S.Y., Park S., Kwon Y. (2019). Recent therapeutic trends and promising targets in triple negative breast cancer. Pharmacol. Ther..

[100] NIH BRCA Mutations: Cancer Risk and Genetic Testing. https://www.cancer.gov/about-cancer/causes-prevention/genetics/brca-fact-sheet#r2.

[101] Cruz C., Castroviejo-Bermejo M., Gutiérrez-Enríquez S., Llop-Guevara A., Ibrahim Y.H., Gris-Oliver A., Bonache S., Morancho B., Bruna A., Rueda O.M. (2018). RAD51 foci as a functional biomarker of homologous recombination repair and PARP inhibitor resistance in germline BRCA-mutated breast cancer. Ann. Oncol..

[102] Roy R., Chun J., Powell S.N. (2011). BRCA1 and BRCA2: Different roles in a common pathway of genome protection. Nat. Rev. Cancer.

[103] Morales J., Li L., Fattah F.J., Dong Y., Bey E.A., Patel M., Gao J., Boothman D.A. (2014). Review of poly (ADP-ribose) polymerase (PARP) mechanisms of action and rationale for targeting in cancer and other diseases. Crit. Rev. Eukaryot Gene Expr..

[104] Caulfield S.E., Davis C.C., Byers K.F. (2019). Olaparib: A Novel Therapy for Metastatic Breast Cancer in Patients With a BRCA1/2 Mutation. J. Adv. Pract. Oncol..

[105] NIH (2020). BRCA Mutation:PARP Inhibitor.

[106] Litton J.K., Rugo H.S., Ettl J., Hurvitz S.A., Gonçalves A., Lee K.-H., Fehrenbacher L., Yerushalmi R., Mina L.A., Martin M. (2018). Talazoparib in Patients with Advanced Breast Cancer and a Germline BRCA Mutation. N. Engl. J. Med..

[107] Keung M.Y.T., Wu Y., Vadgama J.V. (2019). PARP Inhibitors as a Therapeutic Agent for Homologous Recombination Deficiency in Breast Cancers. J. Clin. Med..

[108] Lim E., Johnson S.F., Geyer M., Serra V., Shapiro G.I. (2017). Sensitizing HR-proficient cancers to PARP inhibitors. Mol. Cell Oncol..

[109] Liu X., Kantarjian H., Plunkett W. (2012). Sapacitabine for cancer. Expert Opin Investig Drugs..

[110] Keenan T., Liu D., Elmarakeby H., Stover D., Kochupurakkal B., Tracy A., Danielczyk E., Anderson L., Andrews C., Reardon B. (2019). Abstract CT050: Expansion cohort of Phase I study of oral sapacitabine and oral seliciclib in patients with metastatic breast cancer and *BRCA1/2* mutations. J. Cancer Res..

[111] Jette N.R., Kumar M., Radhamani S., Arthur G., Goutam S., Yip S., Kolinsky M., Williams G.J., Bose P., Lees-Miller S.P. (2020). ATM-Deficient Cancers Provide New Opportunities for Precision Oncology. Cancers.

[112] Wengner A.M., Siemeister G., Lücking U., Lefranc J., Wortmann L., Lienau P., Bader B., Bömer U., Moosmayer D., Eberspächer U. (2020). The Novel ATR Inhibitor BAY 1895344 Is Efficacious as Monotherapy and Combined with DNA Damage–Inducing or Repair–Compromising Therapies in Preclinical Cancer Models. J. Mol. Cancer Ther..

[113] ACS *Key Statistic of Lung Cancer*; ACS. www.cancer.org/cancer/lung-cancer/about/key-statistics.html.

[114] ALA *Lung Cancer Facts Sheet*; ALA. https://www.lung.org/lung-health-diseases/lung-disease-lookup/lung-cancer/resource-library.

[115] ACS *About Lung Cancer*; ACS. www.cancer.org/cancer/lung-cancer/about/what-is.html.

[116] Horita N., Nagashima A., Nakashima K., Shibata Y., Ito K., Goto A., Yamanaka T., Kaneko T. (2017). The best platinum regimens for chemo-naive incurable non-small cell lung cancer: Network meta-analysis. Sci. Rep..

[117] Horita N., Yamamoto M., Sato T., Tsukahara T., Nagakura H., Tashiro K., Shibata Y., Watanabe H., Nagai K., Inoue M. (2015). Topotecan for Relapsed Small-cell Lung Cancer: Systematic Review and Meta-Analysis of 1347 Patients. Sci. Rep..

[118] Chan B.A., Coward J.I. (2013). Chemotherapy advances in small-cell lung cancer. J. Thorac Dis..

[119] Yuan M., Huang L.-L., Chen J.-H., Wu J., Xu Q. (2019). The emerging treatment landscape of targeted therapy in non-small-cell lung cancer. Signal Transduct. Target. Ther..

[120] FDA *FDA Approves First Blood Test to Detect Gene Mutation Associated with Non-Small Cell Lung Cancer*; FDA: 2016. https://www.fda.gov/news-events/press-announcements/fda-approves-first-blood-test-detect-gene-mutation-associated-non-small-cell-lung-cancer.

[121] Schulze A.B., Evers G., Kerkhoff A., Mohr M., Schliemann C., Berdel W.E., Schmidt L.H. (2019). Future Options of Molecular-Targeted Therapy in Small Cell Lung Cancer. Cancers.

[122] Owonikoko T.K., Niu H., Nackaerts K., Csoszi T., Ostoros G., Mark Z., Baik C., Joy A.A., Chouaid C., Jaime J.C. (2020). Randomized Phase II Study of Paclitaxel plus Alisertib versus Paclitaxel plus Placebo as Second-Line Therapy for SCLC: Primary and Correlative Biomarker Analyses. J. Thorac. Oncol..

[123] Atal S., Asokan P., Jhaj R. (2020). Recent advances in targeted small-molecule inhibitor therapy for non-small-cell lung cancer—An update. J. Clin. Pharm..

[124] FDA *FDA Approves First Targeted Therapy to Treat Aggressive Form of Lung Cancer*; FDA: 2020. https://www.fda.gov/news-events/press-announcements/fda-approves-first-targeted-therapy-treat-aggressive-form-lung-cancer.

[125] FDA *FDA Approves Pralsetinib for Lung Cancer with RET Gene Fusions*; FDA: 2020. https://www.fda.gov/drugs/resources-information-approved-drugs/fda-approves-pralsetinib-lung-cancer-ret-gene-fusions#:~:text=FDA%20approves%20pralsetinib%20for%20lung%20cancer%20with%20RET,(NSCLC)%20as%20detected%20by%20an%20FDA%20approved%20test.

[126] FDA *FDA Approves Selpercatinib for Lung and Thyroid Cancers with RET Gene Mutations Or Fusions*; FDA: 2020. https://www.fda.gov/drugs/drug-approvals-and-databases/fda-approves-selpercatinib-lung-and-thyroid-cancers-ret-gene-mutations-or-fusions.

[127] FDA *FDA Approves Nivolumab Plus Ipilimumab for First-Line mNSCLC (PD-L1 Tumor Expression ≥1%)*; FDA: 2020. https://www.fda.gov/drugs/drug-approvals-and-databases/fda-approves-nivolumab-plus-ipilimumab-first-line-mnsclc-pd-l1-tumor-expression-1.

[128] FDA *FDA Approves Nivolumab Plus Ipilimumab and Chemotherapy for First-Line Treatment of Metastatic NSCLC*; FDA: 2020. https://www.fda.gov/drugs/drug-approvals-and-databases/fda-approves-nivolumab-plus-ipilimumab-and-chemotherapy-first-line-treatment-metastatic-nsclc.

[129] Antonia S.J., Villegas A., Daniel D., Vicente D., Murakami S., Hui R., Kurata T., Chiappori A., Lee K.H., de Wit M. (2018). Overall Survival with Durvalumab after Chemoradiotherapy in Stage III NSCLC. N. Engl. J. Med..

[130] FDA *Pembrolizumab (Keytruda) 5-10-2017*. FDA: 2017. https://www.fda.gov/drugs/resources-information-approved-drugs/pembrolizumab-keytruda-5-10-2017.

[131] FDA *FDA Approves Atezolizumab with Chemotherapy and Bevacizumab for First-Line Treatment of Metastatic Non-Squamous NSCLC*; FDA: 2018. https://www.fda.gov/drugs/fda-approves-atezolizumab-chemotherapy-and-bevacizumab-first-line-treatment-metastatic-non-squamous.

[132] FDA *FDA Approves Atezolizumab with Nab-Paclitaxel and Carboplatin for Metastatic NSCLC without EGFR/ALK Aberrations*; FDA: 2019. https://www.fda.gov/drugs/resources-information-approved-drugs/fda-approves-atezolizumab-nab-paclitaxel-and-carboplatin-metastatic-nsclc-without-egfralk.

[133] FDA *FDA Expands Pembrolizumab Indication for First-Line Treatment of NSCLC (TPS ≥1%)*; FDA: 2019. https://www.fda.gov/drugs/fda-expands-pembrolizumab-indication-first-line-treatment-nsclc-tps-1.

[134] FDA *FDA Approves Atezolizumab for First-Line Treatment of Metastatic NSCLC with High PD-L1 Expression*; FDA: 2020. https://www.fda.gov/drugs/resources-information-approved-drugs/fda-approves-atezolizumab-first-line-treatment-metastatic-nsclc-high-pd-l1-expression.

[135] Horn L., Mansfield A.S., Szczesna A., Havel L., Krzakowski M., Hochmair M.J., Huemer F., Losonczy G., Johnson M.L., Nishio M. (2018). First-Line Atezolizumab plus Chemotherapy in Extensive-Stage Small-Cell Lung Cancer. N. Engl. J. Med..

[136] FDA *FDA Approves Durvalumab for Extensive-Stage Small Cell Lung Cancer*; FDA: 2020. https://www.fda.gov/drugs/resources-information-approved-drugs/fda-approves-durvalumab-extensive-stage-small-cell-lung-cancer#:~:text=FDA%20approves%20durvalumab%20for%20extensive-stage%20small%20cell%20lung,patients%20with%20extensive-stage%20small%20cell%20lung%20cancer%20(ES-SCLC).

[137] FDA *FDA Grants Accelerated Approval to Lurbinectedin for Metastatic Small Cell Lung Cancer*; FDA: 2020. https://www.fda.gov/drugs/drug-approvals-and-databases/fda-grants-accelerated-approval-lurbinectedin-metastatic-small-cell-lung-cancer.

[138] Hur J.Y., Ku B.M., Shim J.H., Jung H.A., Sun J.-M., Lee S.-H., Ahn J.S., Park K., Ahn M.-J. (2020). Characteristics and Clinical Outcomes of Non-small Cell Lung Cancer Patients in Korea With MET Exon 14 Skipping. In Vivo.

[139] FDA *FDA Approves Ramucirumab Plus Erlotinib for First-Line Metastatic NSCLC*; FDA: 2020. https://www.fda.gov/drugs/drug-approvals-and-databases/fda-approves-ramucirumab-plus-erlotinib-first-line-metastatic-nsclc.

[140] Yang M., Xu X., Cai J., Ning J., Wery J.P., Li Q.-X. (2016). NSCLC harboring EGFR exon-20 insertions after the regulatory C-helix of kinase domain responds poorly to known EGFR inhibitors. Int. J. Cancer.

[141] Takeda (2020). Takeda Announces U.S. FDA Breakthrough Therapy Designation for Mobocertinib (TAK-788) for the Treatment of NSCLC Patients with EGFR Exon 20 Insertion Mutations.

[142] NIH *Liquid BIopsies in Patients Presenting Non-small Cell Lung Cancer (LIBIL)*; NIH: 2020. https://clinicaltrials.gov/ct2/show/NCT02511288.

[143] Podar K., Tai Y.T., Hideshima T., Vallet S., Richardson P.G., Anderson K.C. (2009). Emerging therapies for multiple myeloma. Expert Opin. Emerg. Drugs.

[144] Bates S.E. (2016). Multiple Myeloma: Multiplying Therapies. J. Clin. Cancer Res..

[145] Kapoor P., Rajkumar S.V., Dispenzieri A., Gertz M.A., Lacy M.Q., Dingli D., Mikhael J.R., Roy V., Kyle R.A., Greipp P.R. (2011). Melphalan and prednisone versus melphalan, prednisone and thalidomide for elderly and/or transplant ineligible patients with multiple myeloma: A meta-analysis. Leukemia.

[146] Rajkumar S.V. (2009). Multiple myeloma. Curr. Probl. Cancer.

[147] Rajan A.M., Rajkumar S.V. (2015). Interpretation of cytogenetic results in multiple myeloma for clinical practice. Blood Cancer J..

[148] Kumar S.K., Rajkumar S.V. (2018). The multiple myelomas—Current concepts in cytogenetic classification and therapy. Nat. Rev. Clin. Oncol..

[149] Korde N., Kristinsson S.Y., Landgren O. (2011). Monoclonal gammopathy of undetermined significance (MGUS) and smoldering multiple myeloma (SMM): Novel biological insights and development of early treatment strategies. Blood.

[150] Rajkumar S.V., Dimopoulos M.A., Palumbo A., Blade J., Merlini G., Mateos M.V., Kumar S., Hillengass J., Kastritis E., Richardson P. (2014). International Myeloma Working Group updated criteria for the diagnosis of multiple myeloma. Lancet Oncol..

[151] Rajkumar S.V. (2016). Myeloma today: Disease definitions and treatment advances. Am. J. Hematol..

[152] Kyle R.A., Rajkumar S.V. (2008). Multiple myeloma. Blood.

[153] Holstein S.A., McCarthy P.L. (2017). Immunomodulatory Drugs in Multiple Myeloma: Mechanisms of Action and Clinical Experience. Drugs.

[154] Chang X., Zhu Y., Shi C., Stewart A.K. (2013). Mechanism of immunomodulatory drugs’ action in the treatment of multiple myeloma. Acta Biochim. Biophys. Sin..

[155] FDA *Lenalidomide (Revlimid)*; FDA: 2017. https://www.fda.gov/drugs/resources-information-approved-drugs/lenalidomide-revlimid.

[156] Mogollón P., Díaz-Tejedor A., Algarín E.M., Paíno T., Garayoa M., Ocio E.M. (2019). Biological Background of Resistance to Current Standards of Care in Multiple Myeloma. Cells.

[157] Mohan M., Matin A., Davies F.E. (2017). Update on the optimal use of bortezomib in the treatment of multiple myeloma. Cancer Manag. Res..

[158] Cavo M., Pantani L., Pezzi A., Petrucci M.T., Patriarca F., Di Raimondo F., Marzocchi G., Galli M., Montefusco V., Zamagni E. (2015). Bortezomib-thalidomide-dexamethasone (VTD) is superior to bortezomib-cyclophosphamide-dexamethasone (VCD) as induction therapy prior to autologous stem cell transplantation in multiple myeloma. Leukemia.

[159] Mai E.K., Bertsch U., Dürig J., Kunz C., Haenel M., Blau I.W., Munder M., Jauch A., Schurich B., Hielscher T. (2015). Phase III trial of bortezomib, cyclophosphamide and dexamethasone (VCD) versus bortezomib, doxorubicin and dexamethasone (PAd) in newly diagnosed myeloma. Leukemia.

[160] Rosinol Dachs L., Hebraud B., Oriol A., Colin A.-L., Rios R., Hulin C., Blanchard M.J., Caillot D., Sureda A., Hernández M.T. (2018). Integrated Analysis of Randomized Controlled Trials Evaluating Bortezomib + Lenalidomide + Dexamethasone or Bortezomib + Thalidomide + Dexamethasone Induction in Transplant-Eligible Newly Diagnosed Multiple Myeloma. Blood.

[161] Moreau P., Masszi T., Grzasko N., Bahlis N.J., Hansson M., Pour L., Sandhu I., Ganly P., Baker B.W., Jackson S.R. (2016). Oral Ixazomib, Lenalidomide, and Dexamethasone for Multiple Myeloma. N. Engl. J. Med..

[162] FDA *Drug Trials Snapshot: FARYDAK (Panobinostat)*; FDA: 2015. https://www.fda.gov/drugs/drug-approvals-and-databases/drug-trials-snapshot-farydak-panobinostat.

[163] NIH *Panobinostat, Carfilzomib, and Dexamethasone in Patients With Relapsed or Refractory Multiple Myeloma*; NIH: 2020. https://clinicaltrials.gov/ct2/show/NCT03256045.

[164] Paul B., Lipe B., Ocio E.M., Usmani S.Z. (2019). Induction Therapy for Newly Diagnosed Multiple Myeloma. Am. Soc. Clin. Oncol. Educ. Book.

[165] Fancher K.M., Bunk E.J. (2016). Elotuzumab: The First Monoclonal Antibody for the Treatment of Multiple Myeloma. J. Adv. Pr. Oncol..

[166] Gormley N.J., Ko C.W., Deisseroth A., Nie L., Kaminskas E., Kormanik N., Goldberg K.B., Farrell A.T., Pazdur R. (2017). FDA Drug Approval: Elotuzumab in Combination with Lenalidomide and Dexamethasone for the Treatment of Relapsed or Refractory Multiple Myeloma. Clin. Cancer Res..

[167] Hori M., Takezako N., Sunami K., Ito S., Kuroda J., Popa-McKiver M., Jou Y.-M., Shelat S.G., Miyoshi M., Suzuki K. (2018). Elotuzumab Plus Pomalidomide/Dexamethasone for the Treatment of Relapsed/Refractory Multiple Myeloma: Japanese Subanalysis of the Randomized Phase 2 Eloquent-3 Study. Blood.

[168] Laubach J., Nooka A.K., Cole C., O’Donnell E., Vij R., Usmani S.Z., Orloff G.J., Richter J.R., Redd R., DiPietro H. (2017). An open-label, single arm, phase IIa study of bortezomib, lenalidomide, dexamethasone, and elotuzumab in newly diagnosed multiple myeloma. J. Clin. Oncol..

[169] De Weers M., Tai Y.-T., van der Veer M.S., Bakker J.M., Vink T., Jacobs D.C.H., Oomen L.A., Peipp M., Valerius T., Slootstra J.W. (2011). Daratumumab, a Novel Therapeutic Human CD38 Monoclonal Antibody, Induces Killing of Multiple Myeloma and Other Hematological Tumors. J. Immunol..

[170] Htut T., Thein K., Lawrie A., Tighe J., Preston G. (2020). Efficacy of daratumumab combination regimen in patients with multiple myeloma: A combined analysis of phase III randomized controlled trials. eJHaem.

[171] Xu X.S., Moreau P., Usmani S.Z., Lonial S., Jakubowiak A., Oriol A., Krishnan A., Bladé J., Luo M., Sun Y.N. (2020). Split First Dose Administration of Intravenous Daratumumab for the Treatment of Multiple Myeloma (MM): Clinical and Population Pharmacokinetic Analyses. Adv. Ther..

[172] FDA *FDA Approves Daratumumab and Hyaluronidase-Fihj for Multiple Myeloma*; FDA: 2020. https://www.fda.gov/drugs/drug-approvals-and-databases/fda-approves-daratumumab-and-hyaluronidase-fihj-multiple-myeloma.

[173] Abdallah N., Kumar S.K. (2019). Daratumumab in untreated newly diagnosed multiple myeloma. Adv. Hematol..

[174] Palumbo A., Dimopoulos M.A., Reece D.E., Sonneveld P., Spencer A., Chanan-Khan A.A.A., Goldschmidt H., Yeh H., Schecter J.M., Qin X. (2015). Twin randomized studies of daratumumab (DARA; D) plus standard of care (lenalidomide/dexamethasone or bortezomib/dexamethasone [DRd or DVd]) versus Rd or Vd alone in relapsed or refractory multiple myeloma (MM): 54767414MMY3003 (Pollux) and 54767414MMY3004 (Castor). J. Clin. Oncol..

[175] Chari A., Suvannasankha A., Fay J.W., Arnulf B., Kaufman J.L., Ifthikharuddin J.J., Weiss B.M., Krishnan A., Lentzsch S., Comenzo R. (2017). Daratumumab plus pomalidomide and dexamethasone in relapsed and/or refractory multiple myeloma. Blood.

[176] Moreau P., Attal M., Hulin C., Arnulf B., Belhadj K., Benboubker L., Béné M.C., Broijl A., Caillon H., Caillot D. (2019). Bortezomib, thalidomide, and dexamethasone with or without daratumumab before and after autologous stem-cell transplantation for newly diagnosed multiple myeloma (CASSIOPEIA): A randomised, open-label, phase 3 study. Lancet.

[177] FDA *FDA Approves Carfilzomib and Daratumumab with Dexamethasone for Multiple Myeloma*; FDA: 2020. https://www.fda.gov/drugs/drug-approvals-and-databases/fda-approves-carfilzomib-and-daratumumab-dexamethasone-multiple-myeloma.

[178] Moreno L., Pérez C., Zabaleta A., Manrique I., Alignani D., Ajona D., Blanco L., Lasa M., Maiso P., Rodriguez I. (2019). The Mechanism Of Action Of The Anti-CD38 Monoclonal Antibody Isatuximab In Multiple Myelmoa. J. Clin. Cancer Res..

[179] FDA *FDA Approves Isatuximab-Irfc for Multiple Myeloma*; FDA: 2020. https://www.fda.gov/drugs/development-approval-process-drugs/fda-approves-isatuximab-irfc-multiple-myeloma#:~:text=On%20March%202%2C%202020%2C%20the,lenalidomide%20and%20a%20proteasome%20inhibitor.

[180] Orlowski R.Z., Goldschmidt H., Cavo M., Martin T.G., Paux G., Oprea C., Facon T. (2018). Phase III (IMROZ) study design: Isatuximab plus bortezomib (V), lenalidomide (R), and dexamethasone (d) vs VRd in transplant-ineligible patients (pts) with newly diagnosed multiple myeloma (NDMM). J. Clin. Oncol..

[181] Raab M.S., Engelhardt M., Blank A., Goldschmidt H., Agis H., Blau I.W., Einsele H., Ferstl B., Schub N., Röllig C. (2020). MOR202, a novel anti-CD38 monoclonal antibody, in patients with relapsed or refractory multiple myeloma: A first-in-human, multicentre, phase 1-2a trial. Lancet Haematol..

[182] Podar K., Shah J., Chari A., Richardson P.G., Jagannath S. (2020). Selinexor for the treatment of multiple myeloma. Expert Opin. Pharm..

[183] Ghobrial I.M., Vij R., Siegel D., Badros A., Kaufman J., Raje N., Jakubowiak A., Savona M.R., Obreja M., Berdeja J.G. (2019). A Phase Ib/II Study of Oprozomib in Patients with Advanced Multiple Myeloma and Waldenström Macroglobulinemia. J. Clin. Cancer Res..

[184] Spencer A., Harrison S., Zonder J., Badros A., Laubach J., Bergin K., Khot A., Zimmerman T., Chauhan D., Levin N. (2018). A phase 1 clinical trial evaluating marizomib, pomalidomide and low-dose dexamethasone in relapsed and refractory multiple myeloma (NPI-0052-107): Final study results. Br. J. Haematol..

[185] NIH *National Center for Biotechnology Information. PubChem Database. Melphalan Flufenamide, CID=9935639*; NIH: 2020. https://pubchem.ncbi.nlm.nih.gov/compound/Melphalan-flufenamide.

[186] Oncopeptides *Melflufen–Investigational Drug*; Oncopeptides: 2020. https://www.oncopeptides.com/en/innovation/investigational-drug.

[187] Kumar S., Harrison S.J., Cavo M., Rubia J.D.L., Popat R., Gasparetto C., Hungria V., Salwender H., Suzuki K., Kim I. (2020). Updated results from BELLINI, a phase III study of venetoclax or placebo in combination with bortezomib and dexamethasone in relapsed/refractory multiple myeloma. J. Clin. Oncol..

[188] AbbVie (2019). AbbVie Announces US FDA Lifts Partial Clinical Hold on Phase 3 Study of Venetoclax in Patients with Multiple Myeloma Positive for the t(11;14) Genetic Abnormality.

[189] FDA *FDA Warns about the Risks Associated with the Investigational use of Venclexta in Multiple Myeloma*; FDA: 2019. https://www.fda.gov/drugs/drug-safety-and-availability/fda-warns-about-risks-associated-investigational-use-venclexta-multiple-myeloma#:~:text=Venclexta%20is%20safe%20and%20effective,these%20trials%20after%20they%20reconsent.

[190] Cho S.F., Anderson K.C., Tai Y.T. (2018). Targeting B Cell Maturation Antigen (BCMA) in Multiple Myeloma: Potential Uses of BCMA-Based Immunotherapy. Front. Immunol..

[191] Mullard A. (2019). The BCMA bonanza. Nat. Rev. Drug Discov..

[192] Tai Y.-T., Mayes P.A., Acharya C., Zhong M.Y., Cea M., Cagnetta A., Craigen J., Yates J., Gliddon L., Fieles W. (2014). Novel anti–B-cell maturation antigen antibody-drug conjugate (GSK2857916) selectively induces killing of multiple myeloma. Blood.

[193] FDA *FDA Granted Accelerated Approval to Belantamab Mafodotin-Blmf for Multiple Myeloma*; FDA: 2020. https://www.fda.gov/drugs/drug-approvals-and-databases/fda-granted-accelerated-approval-belantamab-mafodotin-blmf-multiple-myeloma.

[194] Trudel S., Lendvai N., Popat R., Voorhees P.M., Reeves B., Libby E.N., Richardson P.G., Hoos A., Gupta I., Bragulat V. (2019). Antibody–drug conjugate, GSK2857916, in relapsed/refractory multiple myeloma: An update on safety and efficacy from dose expansion phase I study. Blood Cancer J..

[195] GSK GSK Announces Further Positive Data from DREAMM-1 Study of anti-BCMA Antibody-Drug Conjugate in Patients with Relapsed/Refractory Multiple Myeloma; GSK: 2019. https://www.gsk.com/en-gb/media/press-releases/gsk-announces-further-positive-data-from-dreamm-1-study-of-anti-bcma-antibody-drug-conjugate/.

[196] Xing L., Lin L., Yu T., Li Y., Wen K., Cho S.-F., Hsieh P.A., Kinneer K., Munshi N.C., Anderson K.C. (2019). Anti-Bcma PBD MEDI2228 Combats Drug Resistance and Synergizes with Bortezomib and Inhibitors to DNA Damage Response in Multiple Myeloma. Blood.

[197] Hipp S., Deegen P., Wahl J., Blanset D., Thomas O., Rattel B., Adam P., Friedrich M. (2015). BI 836909, a Novel Bispecific T Cell Engager for the Treatment of Multiple Myeloma Induces Highly Specific and Efficacious Lysis of Multiple Myeloma Cells in Vitro and Shows Anti-Tumor Activity in Vivo. Blood.

[198] Goyos A., Li C.-M., Deegen P., Bogner P., Thomas O., Matthias K., Wahl J., Goldstein R., Coxon A., Balazs M. (2017). Generation of Half-Life Extended Anti-BCMA Bite^®^ Antibody Construct Compatible with Once-Weekly Dosing for Treatment of Multiple Myeloma (MM). Blood.

[199] Usmani S.Z., Mateos M.V., Nahi H., Krishnan A.Y., van de Donk N.W., San Miguel J., Miguel J.S., Oriol A., Rosiñol L., Chari A. (2020). Phase I study of teclistamab, a humanized B-cell maturation antigen (BCMA) x CD3 bispecific antibody, in relapsed/refractory multiple myeloma (R/R MM). J. Clin. Oncol..

[200] Caraccio C., Krishna S., Phillips D.J., Schürch C.M. (2020). Bispecific Antibodies for Multiple Myeloma: A Review of Targets, Drugs, Clinical Trials, and Future Directions. Front. Immunol..

[201] BMS (2019). Bristol-Myers Squibb and bluebird bio Announce Positive Top-line Results from the Pivotal Phase 2 KarMMa Study of Ide-cel in Relapsed and Refractory Multiple Myeloma. Study Met Its Primary Endpoint and Key Secondary Endpoint, Demonstrating Deep and Durable Responses in a Heavily Pre-Treated Multiple Myeloma Patient Population.

[202] BMS (2019). Bluebird Bio and Bristol-Myers Squibb Present Updated Data from Ongoing Phase 1 Study of BCMA-Targeted CAR T Cell Therapy bb21217 in Relapsed/Refractory Multiple Myeloma at 61st ASH Annual Meeting and Exposition.

[203] Berdeja J.G., Madduri D., Usmani S.Z., Singh I., Zudaire E., Yeh T.-M., Allred A.J., Olyslager Y., Banerjee A., Goldberg J.D. (2020). Update of CARTITUDE-1: A phase Ib/II study of JNJ-4528, a B-cell maturation antigen (BCMA)-directed CAR-T-cell therapy, in relapsed/refractory multiple myeloma. J. Clin. Oncol..

[204] Bjorklund C.C., Lu L., Kang J., Hagner P.R., Havens C.G., Amatangelo M., Wang M., Ren Y., Couto S., Breider M. (2015). Rate of CRL4(CRBN) substrate Ikaros and Aiolos degradation underlies differential activity of lenalidomide and pomalidomide in multiple myeloma cells by regulation of c-Myc and IRF4. Blood Cancer J..

[205] Bjorklund C.C., Kang J., Amatangelo M., Polonskaia A., Katz M., Chiu H., Couto S., Wang M., Ren Y., Ortiz M. (2020). Iberdomide (CC-220) is a potent cereblon E3 ligase modulator with antitumor and immunostimulatory activities in lenalidomide- and pomalidomide-resistant multiple myeloma cells with dysregulated CRBN. Leukemia.

[206] Hansen J.D., Correa M., Nagy M.A., Alexander M., Plantevin V., Grant V., Whitefield B., Huang D., Kercher T., Harris R. (2020). Discovery of CRBN E3 Ligase Modulator CC-92480 for the Treatment of Relapsed and Refractory Multiple Myeloma. J. Med. Chem..

[207] Wong L., Narla R.K., Leisten J., Bauer D., Groza M., Gaffney B., Havens C.G., Choi J., Houston J., Lopez-Girona A. (2019). CC-92480, a Novel Cereblon E3 Ligase Modulator, Is Synergistic with Dexamethasone, Bortezomib, and Daratumumab in Multiple Myeloma. Blood.

[208] Amatangelo M., Bjorklund C.C., Kang J., Polonskaia A., Viswanatha S., Thakurta A. (2018). Iberdomide (CC-220) Has Synergistic Anti-Tumor and Immunostimulatory Activity Against Multiple Myeloma in Combination with Both Bortezomib and Dexamethasone, or in Combination with Daratumumab in Vitro. Blood.

[209] NIH A Study to Determine Dose, Safety, Tolerability and Efficacy of CC-220 Monotherapy, and in Combination with Other Treatments in Subjects with Multiple Myeloma; NIH: 2020. https://clinicaltrials.gov/ct2/show/NCT02773030.

[210] Pestana R.C., Sen S., Hobbs B.P., Hong D.S. (2020). Histology-agnostic drug development - Considering issues beyond the tissue. Nat. Rev. Clin. Oncol..

[211] Le D.T., Durham J.N., Smith K.N., Wang H., Bartlett B.R., Aulakh L.K., Lu S., Kemberling H., Wilt C., Luber B.S. (2017). Mismatch repair deficiency predicts response of solid tumors to PD-1 blockade. Science.

[212] Garber K. (2018). Tissue-agnostic cancer drug pipeline grows, despite doubts. Nat. Rev. Drug Discov..

[213] Chu P., Batson S., Hodgson M., Mitchell C.R., Steenrod A. (2020). Systematic review of neurotrophic tropomyosin-related kinase inhibition as a tumor-agnostic management strategy. Future Oncol..

[214] Li I.W., Krishnamurthy N., Wei G., Li G. (2020). Opportunities and challenges in developing tissue-agnostic anti-cancer drugs. J. Cancer Metastasis Treat.

[215] Pessoa L.S., Heringer M., Ferrer V.P. (2020). ctDNA as a cancer biomarker: A broad overview. Crit. Rev. Oncol. Hematol..

[216] Liu M.C., Oxnard G.R., Klein E.A., Swanton C., Seiden M. (2020). Sensitive and specific multi-cancer detection and localization using methylation signatures in cell-free DNA. Ann. Oncol..

[217] Fiala C., Diamandis E.P. (2020). A multi-cancer detection test: Focus on the positive predictive value. Ann. Oncol..

[218] Taylor W.C. (2020). Comment on ‘Sensitive and specific multi-cancer detection and localization using methylation signatures in cell-free DNA’ by M. C. Liu et al. Ann. Oncol..

[219] Chen X., Gole J., Gore A., He Q., Lu M., Min J., Yuan Z., Yang X., Jiang Y., Zhang T. (2020). Non-invasive early detection of cancer four years before conventional diagnosis using a blood test. Nat. Commun..

