# Supplementary material for: Therapeutic Advances in Oncology
Source: Int J Mol Sci. 2021 Feb 18;22(4):2008. doi: 10.3390/ijms22042008 (PMC7922397; doi:10.3390/ijms22042008)
Supplement: Supplementary file 1 [file ijms-22-02008-s001.pdf]

Table S1. Novel drug approvals including cell/gene therapy summarized by therapeutic areas (2015–2020).

	Diabetes	Immunology	Neurology	Oncology
2015	1	2	3	15
2016	1	3	4	6
2017	2	5	6	14
2018	0	6	8	17
2019	0	3	9	11
9/2020	0	2	9	14
Total	4	21	39	77

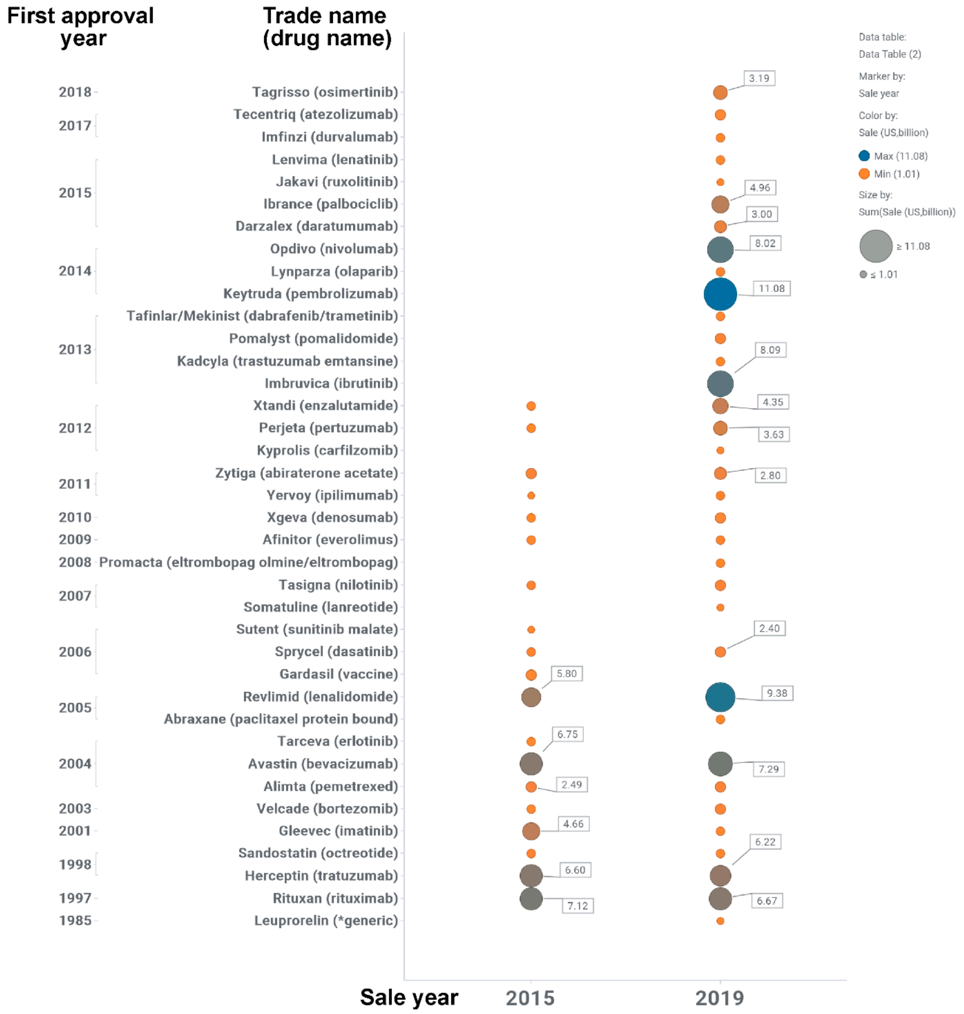

Figure S1. Comparison of global therapeutic sales of the top selling drugs (>1 billion USD) in oncology in 2015 vs. 2019. Top twenty sales are labeled. The first approval year for each drug is listed on the far left, and the drugs are ordered by the approval year from the most recent (top) to earlier (bottom). Data extracted from https://njardarson.lab.arizona.edu/content/top-pharmaceuticals-poster.
